# Description of six new species of the subgenus Panophrys within the genus *Megophrys* (Anura, Megophryidae) from southeastern China based on molecular and morphological data

**DOI:** 10.3897/zookeys.851.29107

**Published:** 2019-06-03

**Authors:** Jian Wang, Zhi-Tong Lyu, Zu-Yao Liu, Cheng-Kai Liao, Zhao-Chi Zeng, Jian Zhao, Yu-Long Li, Ying-Yong Wang

**Affiliations:** 1 State Key Laboratory of Biocontrol / The Museum of Biology, School of Life Sciences, Sun Yat-sen University, Guangzhou 510275, China Sun Yat-sen University Guangzhou China; 2 Jiulianshan National Nature Reserve, Longnan County, Jiangxi 341700, China Jiulianshan National Nature Reserve Jiangxi China

**Keywords:** Conservation, *
Megophrys
*, southeastern China, species diversity, subgenus *Panophrys*, speciation, biogeography

## Abstract

The diversity of the subgenus Panophrys within the genus *Megophrys* has been revealed to be extremely underestimated from southeastern China. Herpetological surveys coupled with extensive sampling in a longitudinal mountain belt located in southeastern China resulted in the discoveries of six new species of the subgenus Panophrys. Furthermore, the new discoveries support the findings of “micro-endemism”, “sympatric phenomenon” and “sympatric but distant phylogenetically” which appear to be common among *Panophrys* species, and also indicates that the Asian horned toads would be good candidates for studies on speciation and biogeography, and additionally emphasizes the conservation difficulties of these toads.

## Introduction

The Asian horned toads (*Megophrys*) comprise 85 recognized species which were previously classified in the subfamily Megophryinae (Frost 2019). They are widespread in montane forest area in tropical and subtropical Asia, including southern mainland China, southern and eastern Himalayas, across Indochina to Malay, to the islands of the Sunda Shelf and the Philippines (Chen et al. 2017; Deuti et al. 2017; Mahony et al. 2017; Mahony et al. 2018; Li et al. 2018; Liu et al. 2018; Munir et al. 2018; Messenger et al. 2019; Tapley et al. 2018; Frost 2019). As a consequence of both morphological similarity among species and the complex patterns of genetic divergence, the taxonomy of these toads always has been controversial. Although several researchers have proposed different taxonomic schemes in recent decades (Dubois 1987; Rao and Yang 1997; Dubois and Ohler 1998; Jiang et al. 2003; Zheng et al. 2004; Frost et al. 2006; Li and Wang 2008; Fei et al. 2009; Fei and Ye 2016; Chen et al. 2017; Mahony et al. 2017), the debate remains. Based on a large-scale molecular analysis, Chen et al. (2017) considered that subfamily Megophryinae is composed of five genera, namely *Atympanophrys* Tian & Hu, 1983, *Brachytarsophrys* Tian & Hu, 1983, *Megophrys* Kuhl & Van Hasselt, 1822, *Ophryophryne* Boulenger, 1903 and *Xenophrys* Günther, 1864. Almost at the same time, based on the integrative analysis with phylogeny and morphological examination, Mahony et al. (2017) treated the entire subfamily Megophryinae as a single genus *Megophrys* and divided it into seven subgenera, i.e. *Atympanophrys*, *Brachytarsophrys*, *Megophrys*, *Ophryophryne*, *Panophrys* Rao & Yang, 1997, *Pelobatrachus* Beddard, 1908 and *Xenophrys*, and 25 known species were placed in the subgenus Panophrys. Subsequently, Liu et al. (2018) partially agreed with this taxonomic system based on a substantial study on phylogenetic similarity, and revealed unusually high levels of species diversity in the subgenus Panophrys with a total number of 60 species, 2.4 times of previously known, including 41 unnamed cryptic species and 39 of which were from southeastern China. Therefore, *Panophrys* species diversity from southeastern China is extremely underestimated.

**Figure 1.**
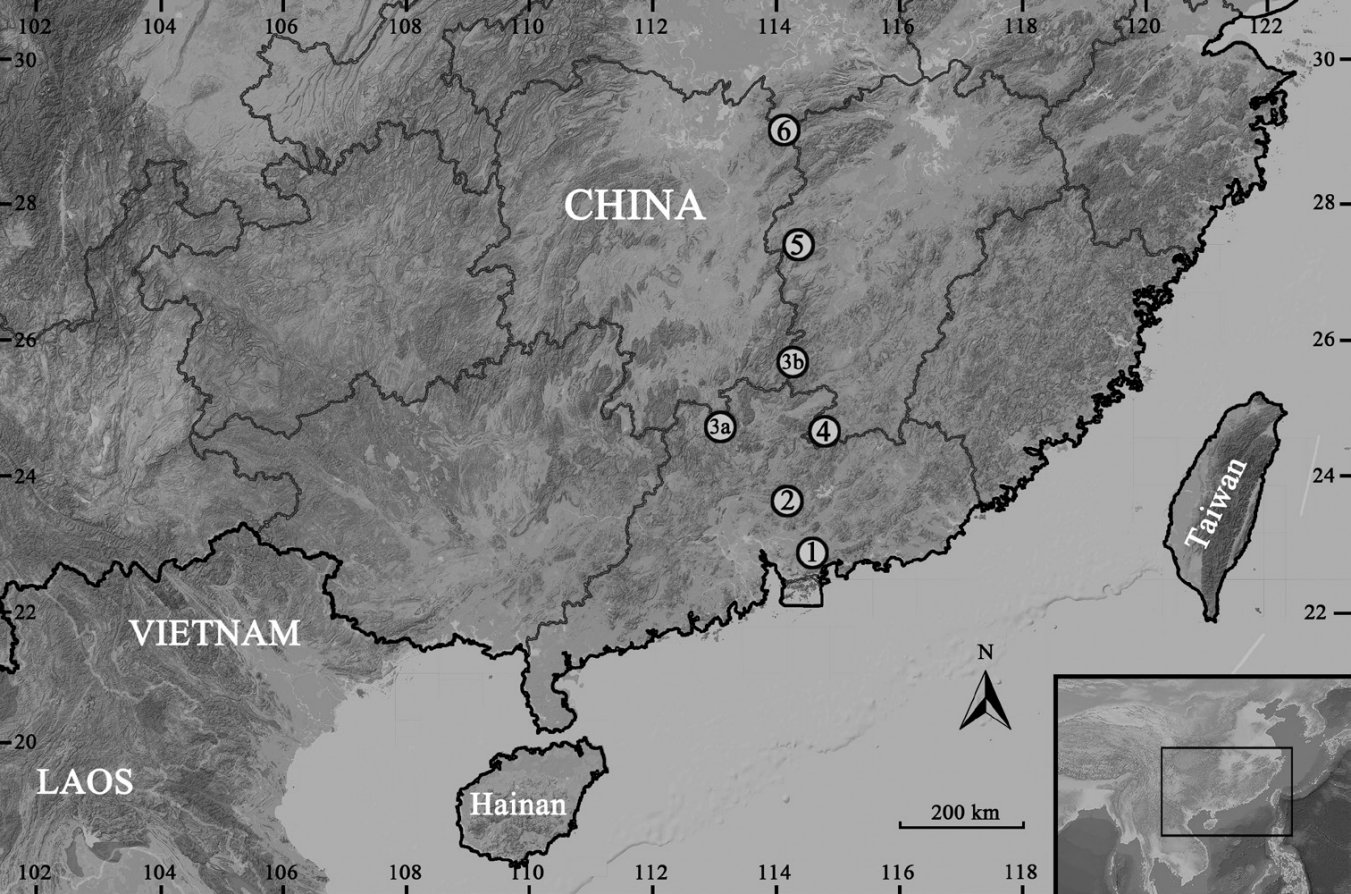
Collection localities of the six new *Megophrys* species in this study: **1** Mt. Yinping in Dongguan City of Guangdong Province, the type locality of *M.dongguanensis* sp. nov. **2** Mt. Nankun in Huizhou City of Guangdong Province, the type locality of *M.nankunensis* sp. nov., and one of the localities of its sympatric species, *M.jiulianensis* sp. nov. **3a** Nanling Nature Reserve in Shaoguan City of Guangdong Province, the type locality of *M.nanlingensis* sp. nov. **3b** Mt. Qiyun in Ganzhou City of Jiangxi Province, the other collection locality of *M.nanlingensis* sp. nov. **4** Mt. Jiulian in Ganzhou City of Jiangxi Province, the type locality of *M.jiulianensis* sp. nov. **5** Yangshimu Scenic Area in Pingxiang City and adjacent Wugongshan Scenic Area in Ji’an City of Jiangxi Province, the type locality of *M.wugongensis* sp. nov. **6** Mt. Mufu in Yueyang City of Hunan Province, the type locality of *M.mufumontana* sp.nov.

In the past years, we have carried out continual herpetological surveys coupled with extensive sampling in a longitudinal mountain belt with a west-east width of 100 km, north-south length of 800 km in the middle of southeastern China, from Hong Kong and Shenzhen in the Pearl River Delta, across the Jiulian Mountains and Luoxiao Mountains, north to the Yangtze River (Fig. 1). The surveys resulted in the discovery of 15 unnamed *Panophrys* species (Liu et al. 2018) and descriptions of 14 new species of amphibians and reptiles, namely *Leptobrachellalaui* (Sung, Yang & Wang, 2014), Megophrys (Brachytarsophrys) popei (Zhao, Yang, Chen, Chen & Wang, 2014), M. (Panophrys) cheni (Wang & Liu, 2014), M. (Pa.) lini (Wang & Yang, 2014), Megophrys (Pa.) jinggangensis (Wang, 2012), *Nidirananankunensis* Lyu, Zeng, Wang, Lin, Liu & Wang, 2017, *Amolopsalbispinus* Sung, Hu, Wang, Liu & Wang, 2016, *Gracixalusjinggangensis* Zeng, Zhao, Chen, Chen, Zhang & Wang, 2017 and *Gr.guangdongensis* Wang, Zeng, Lyu, Liu & Wang, 2018; *Goniurosaurusyingdeensis* Wang, Yang & Cui, 2010, *Go.zhelongi* Wang, Jin, Li & Grismer, 2014, *Takydromusalbomaculosus* Wang, Gong, Liu & Wang, 2017, *Rhabdophisguangdongensis* Zhu, Wang, Takeuchi & Zhao, 2014 and *Opisthotropisshenzhenensis* Wang, Guo, Liu, Lyu, Wang, Luo, Sun & Zhang, 2017.

In the present study, we re-reviewed several species defined by Liu et al. (2018) from this mountain belt based on molecular and morphological data and formally described six new species of *Megophrys*.

## Material and methods

### Sampling

For molecular analysis, a total of 42 samples (17 were attained from GenBank and 25 were new materials in this study) from the collection of unnamed specimens of the subgenus Panophrys, together with 39 samples (37 from GenBank and two new materials) from 21 recognized species of *Panophrys* were used as in-groups in this study. In addition, four samples (all from GenBank) from two recognized species of the subgenus Atympanophrys, four samples (three from GenBank and one new materials) from two recognized species of the subgenus Brachytarsophrys, three samples (one from GenBank and two new materials) from two recognized species of the subgenus Ophryophryne, two samples (all from GenBank) from two recognized species of the subgenus Pelobatrachus, and six samples (five from GenBank and one new materials) of three recognized species of the subgenus Xenophrys were incorporated into our dataset and used as out-groups. Details see Table 1. All muscle samples were preserved in 95% ethanol and stored at -40 °C.

**Table 1.** Localities, voucher information, and GenBank accession numbers for all specimens used in this study.

Subgenus of *Megophrys* s. l.	ID	Species name	Locality	Specimen voucher no.	Genbank Accession No.	
					16S	CO1
*** Panophrys ***	1	*M.dongguanensis* sp. nov.	China: Mt. Yinping, Dongguan City, Guangdong	SYS a001971/CIB110006	MK524097	MK524128
	2	*M.dongguanensis* sp. nov.	China: Mt. Yinping, Dongguan City, Guangdong	SYS a001972	MK524098	MK524129
	3	*M.dongguanensis* sp. nov.	China: Mt. Yinping, Dongguan City, Guangdong	SYS a001973	MH406647	MH406083
	4	*M.dongguanensis* sp. nov.	China: Mt. Yinping, Dongguan City, Guangdong	SYS a001974	MH406648	MH406084
	5	*M.dongguanensis* sp. nov.	China: Mt. Yinping, Dongguan City, Guangdong	SYS a001975	MH406649	MH406085
	6	*M.dongguanensis* sp. nov.	China: Mt. Yinping, Dongguan City, Guangdong	SYS a002007	MH406654	MH406090
	7	*M.jiulianensis* sp. nov.	China: Mt. Jiulian, Ganzhou City, Jiangxi	SYS a002107	MK524099	MK524130
	8	*M.jiulianensis* sp. nov.	China: Mt. Jiulian, Ganzhou City, Jiangxi	SYS a002108	MK524100	MK524131
	9	*M.jiulianensis* sp. nov.	China: Mt. Jiulian, Ganzhou City, Jiangxi	SYS a002109	MK524101	MK524132
	10	*M.jiulianensis* sp. nov.	China: Mt. Jiulian, Ganzhou City, Jiangxi	SYS a004219	MH406791	MH406253
	11	*M.jiulianensis* sp. nov.	China: Mt. Nankun, Huizhou City, Guangdong	SYS a003622	MK524102	MK524133
	12	*M.jiulianensis* sp. nov.	China: Mt. Nankun, Huizhou City, Guangdong	SYS a003623	MK524103	MK524134
	13	*M.mufumontana* sp. nov.	China: Mt. Mufu, Pingjiang County, Hunan	SYS a006390/CIB110012	MK524104	MK524135
	14	*M.mufumontana* sp. nov.	China: Mt. Mufu, Pingjiang County, Hunan	SYS a006391	MK524105	MK524136
	15	*M.mufumontana* sp. nov.	China: Mt. Mufu, Pingjiang County, Hunan	SYS a006392	MK524106	MK524137
	16	*M.mufumontana* sp. nov.	China: Mt. Mufu, Pingjiang County, Hunan	SYS a006419	MK524107	MK524138
	17	*M.nankunensis* sp. nov.	China: Mt. Nankun, Huizhou City, Guangdong	SYS a004498	MK524108	MK524139
	18	*M.nankunensis* sp. nov.	China: Mt. Nankun, Huizhou City, Guangdong	SYS a004499	MK524109	MK524140
	19	*M.nankunensis* sp. nov.	China: Mt. Nankun, Huizhou City, Guangdong	SYS a004500	MK524110	MK524141
	20	*M.nankunensis* sp. nov.	China: Mt. Nankun, Huizhou City, Guangdong	SYS a004501	MH406822	MH406284
	21	*M.nankunensis* sp. nov.	China: Mt. Nankun, Huizhou City, Guangdong	SYS a004502	MH406823	MH406285
	22	*M.nankunensis* sp. nov.	China: Mt. Nankun, Huizhou City, Guangdong	SYS a004503	MH406824	MH406286
*** Panophrys ***	23	*M.nanlingensis* sp. nov.	China: Nanling Nature Reserve, Shaoguan City, Guangdong	SYS a001959	MK524111	MK524142
	24	*M.nanlingensis* sp. nov.	China: Nanling Nature Reserve, Shaoguan City, Guangdong	SYS a001960	MK524112	MK524143
	25	*M.nanlingensis* sp. nov.	China: Nanling Nature Reserve, Shaoguan City, Guangdong	SYS a001964	MH406646	MH406082
	26	*M.nanlingensis* sp. nov.	China: Mt. Qiyun, Chongyi County, Jiangxi	SYS a002334	MH406686	MH406132
	27	*M.nanlingensis* sp. nov.	China: Mt. Qiyun, Chongyi County, Jiangxi	SYS a002356	MK524113	MK524144
	28	*M.nanlingensis* sp. nov.	China: Mt. Qiyun, Chongyi County, Jiangxi	SYS a002357	MH406687	MH406133
	29	*M.nanlingensis* sp. nov.	China: Mt. Qiyun, Chongyi County, Jiangxi	SYS a002358	MH406688	MH406134
	30	*M.wugongensis* sp. nov.	China: Wugongshan Scenic Area, Anfu County, Jiangxi	SYS a002610	MK524114	MK524145
	31	*M.wugongensis* sp. nov.	China: Wugongshan Scenic Area, Anfu County, Jiangxi	SYS a002611	MK524115	MK524146
	32	*M.wugongensis* sp. nov.	China: Yangshimu Scenic Area, Pingxiang City, Jiangxi	SYS a002625	MK524116	MK524147
	33	*M.wugongensis* sp. nov.	China: Wugongshan Scenic Area, Anfu County, Jiangxi	SYS a004777/CIB110011	MK524117	MK524148
	34	*M.wugongensis* sp. nov.	China: Wugongshan Scenic Area, Anfu County, Jiangxi	SYS a004796	MK524118	MK524149
	35	*M.wugongensis* sp. nov.	China: Wugongshan Scenic Area, Anfu County, Jiangxi	SYS a004797	MK524119	MK524150
	36	*M.wugongensis* sp. nov.	China: Wugongshan Scenic Area, Anfu County, Jiangxi	SYS a004798	MK524120	MK524151
	37	*M.wugongensis* sp. nov.	China: Wugongshan Scenic Area, Anfu County, Jiangxi	SYS a004799	MH406852	MH406314
	38	*M.wugongensis* sp. nov.	China: Wugongshan Scenic Area, Anfu County, Jiangxi	SYS a004800	MH406853	MH406315
	39	*M.wugongensis* sp. nov.	China: Wugongshan Scenic Area, Anfu County, Jiangxi	SYS a004801	MH406854	MH406316
	40	*M.wugongensis* sp. nov.	China: Wugongshan Scenic Area, Anfu County, Jiangxi	SYS a004802	MH406855	MH406317
	41	*M.wugongensis* sp. nov.	China: Wugongshan Scenic Area, Anfu County, Jiangxi	SYS a004803	MH406856	MH406318
	42	*M.wugongensis* sp. nov.	China: Wugongshan Scenic Area, Anfu County, Jiangxi	SYS a004804	MK524121	MK524152
	43	* M. acuta *	China: Heishiding Nature Reserve, Zhaoqing City, Guangdong	SYS a001957	KJ579118	MF667898
	44	* M. acuta *	China: Heishiding Nature Reserve, Zhaoqing City, Guangdong	SYS a002159	MF667869	MF667899
*** Panophrys ***	45	* M. binlingensis *	China: Mt. Wawu, Meishan City, Sichuan	SYS a005313	MH406892	MH406354
	46	* M. binlingensis *	China: Mt. Wawu, Meishan City, Sichuan	SYS a005314	MH406893	MH406355
	47	* M. boettgeri *	China: Longhu Forest Station, Shaowu City, Fujian	SYS a004126	MH406785	MH406245
	48	* M. boettgeri *	China: Mt. Wuyi, Fujian	SYS a004150	MF667879	MF667914
	49	* M. brachykolos *	China: Hongkong	SYS a005563	MK524122	MK524153
	50	* M. brachykolos *	China: Hongkong	SYS a005564	MK524123	MK524154
	51	* M. caudoprocta *	China: Mt. Badagongshan, Zhangjiajie City, Hunan	SYS a004281	MH406795	MH406257
	52	* M. caudoprocta *	China: Mt. Badagongshan, Zhangjiajie City, Hunan	SYS a004293	MH406796	MH406258
	53	* M. cheni *	China: Taoyuandong Nature Reserve, Zhuzhou City, Hunan	SYS a002123	KJ560396	MF667904
	54	* M. cheni *	China: Taoyuandong Nature Reserve, Zhuzhou City, Hunan	SYS a002140	MF667872	MF667905
	55	* M. huangshanensis *	China: Mt. Huangshan, Anhui	SYS a002702	MF667882	MF667919
	56	* M. huangshanensis *	China: Mt. Huangshan, Anhui	SYS a002703	MF667883	MF667920
	57	* M. insularis *	China: Nan’ao Island, Guangdong	SYS a002169 (Holotype)	MF667887	MF667924
	58	* M. insularis *	China: Nan’ao Island, Guangdong	SYS a002170	MF667888	MF667925
	59	* M. jingdongensis *	China: Mt. Wuliang, Yunnan	SYS a003928	MH406773	MH406232
	60	* M. jingdongensis *	China: Mt. Wuliang, Yunnan	SYS a003929	MH406774	MH406233
	61	* M. jinggangensis *	China: Mt. Jinggang, Jiangxi	SYS a004028	MH406780	MH406239
	62	* M. jinggangensis *	China: Mt. Sifang, Hengdong County, Hunan	SYS a004825	MH406858	MH406320
	63	* M. kuatunensis *	China: Mt. Wuyi, Jiangxi	SYS a003449	MF667881	MF667916
	64	* M. lini *	China: Nanfengmian Nature Reserve, Jiangxi	SYS a002128	KJ560416	MF667907
	65	* M. lini *	China: Nanfengmian Nature Reserve, Jiangxi	SYS a002381	MF667874	MF667908
	66	* M. minor *	China: Dujiangyan City, Sichuan	SYS a003209	MF667862	MF667891
	67	* M. minor *	China: Dujiangyan City, Sichuan	SYS a003210	MF667863	MF667892
	68	* M. obesa *	China: Heishiding Nature Reserve, Guangdong	SYS a002271	KJ579121	MH406123
	69	* M. obesa *	China: Heishiding Nature Reserve, Guangdong	SYS a005025	MH406868	MH406330
	70	* M. ombrophila *	China: Mt. Wuyi, Fujian	WUYI2015101	KX856397	/
	71	* M. omeimontis *	China: Mt. Laojunshan, Yibin City, Sichuan	SYS a002741	MH406710	MH406162
*** Panophrys ***	72	* M. omeimontis *	China: Hejiang County, Sichuan	SYS a004916	MH406864	MH406326
	73	* M. sangzhiensis *	China: Mt. Badagongshan, Hunan	SYS a004307	MH406798	MH406260
	74	* M. sangzhiensis *	China: Mt. Badagongshan, Hunan	SYS a004313	MH406802	MH406264
	75	* M. spinata *	China: Mt. Leigong, Guizhou	SYS a002226	MH406675	MH406115
	76	* M. spinata *	China: Mt. Leigong, Guizhou	SYS a002227	MH406676	MH406116
	77	* M. tuberogranulatus *	China: Mt. Badagongshan, Hunan	SYS a004310	MH406801	MH406263
	78	* M. wushanensis *	China: Shennongjia Forestry District, Hubei	SYS a003008	MH406732	MH406184
	79	* M. wushanensis *	China: Shennongjia Forestry District, Hubei	SYS a003009	MH406733	MH406185
	80	* M. wuliangshanensis *	China: Mt. Wuliang, Yunnan	SYS a003924	MH406771	MH406230
	81	* M. wuliangshanensis *	China: Mt. Wuliang, Yunnan	SYS a003925	MH406772	MH406231
*** Atympanophrys ***	82	* M. gigantica *	China: Mt. Ailao, Yunnan	SYS a003883	MH406766	MH406225
	83	* M. gigantica *	China: Mt. Wuliang, Yunnan	SYS a003933	MH406775	MH406234
	84	* M. shapingensis *	China: Mt. Wawu, Sichuan	SYS a005310	MH406890	MH406352
	85	* M. shapingensis *	China: Zhaojue County, Sichuan	SYS a005339	MH406897	MH406359
*** Brachytarsophrys ***	86	* M. chuannanensis *	China: Hejiang County, Sichuan	SYS a004926	MH406901	MH406364
	87	* M. chuannanensis *	China: Hejiang County, Sichuan	SYS a004927	MH406902	MH406365
	88	* M. popei *	China: Taoyuandong Nature Reserve, Hunan	SYS a001864	MH406361	KM504256
	89	* M. popei *	China: Mt. Jinggang, Jiangxi	SYS a004209	MK524124	MK524155
*** Ophryophryne ***	90	* M. hansi *	Vietnam: Quang Nam, Tra My District	AMNH 163680	KY022203	/
	91	* M. microstoma *	China: Mt. Wuhuang, Pubei County, Guangxi	SYS a003492	MK524125	MK524156
	92	* M. microstoma *	China: Mt. Wuhuang, Pubei County, Guangxi	SYS a003493	MK524126	MK524157
*** Pelobatrachus ***	93	* M. nasuta *	Malaysia: Sabah, Lahad Datu District	FMNH 231281	KY022186	/
	94	* M. stejnegeri *	Philippines: Mindanao, Bukidnon Province	FMNH 250842	KY022190	/
*** Xenophrys ***	95	* M. glandulosa *	China: Mt. Gaoligong, Yunnan	SYS a003758	MH406755	MH406214
	96	* M. glandulosa *	China: Mt. Gaoligong, Yunnan	SYS a003794	MH406759	MH406218
	97	* M. mangshanensis *	China: Mt. Longtou, Guangdong	SYS a002750	MF667866	MF667895
	98	* M. mangshanensis *	China: Mt. Dayao, Guangxi	SYS a004870	MH406861	MH406323
	99	* M. medogensis *	China: Medog County, Tibet	SYS a002932	MH406725	MH406177
	100	* M. medogensis *	China: Medog County, Tibet	SYS a002933	MK524127	MK524158

### DNA Extraction, PCR and sequencing

Genomic DNA was extracted from muscular tissue using a DNA extraction kit from Tiangen Biotech (Beijing) Co., Ltd. All samples were sequenced for two mitochondrial genes, i.e., partial 16S ribosomal RNA gene (16S) and complete cytochrome C oxidase 1 gene (CO1). Primers used for 16S were L3975 (5’-CGCCTGTTTACCAAAAACAT-3’) and H4551 (5’-CCGGTCTGAACTCAGATCACGT-3’) following Simon et al. (1994), and for CO1 were Chmf4 (5’-TYTCWACWAAYCAYAAAGAYATCGG-3’) and Chmr4 (5’-ACYTCRGGRTGRCCRAARAATCA-3’) following Meyer et al. (2005). PCR amplifications were processed in a 20-reaction volume with the cycling conditions that initial denaturing step at 95 °C for 4 min, 35 cycles of denaturing at 94 °C for 40 s, annealing at 53 °C for 40 s and extending at 72 °C for 1 min, and final extending step of 72 °C for 10 min. PCR products were purified with spin columns. The purified products were sequenced with both forward and reverse primers using BigDye Terminator Cycle Sequencing Kit per the guidelines, on an ABI Prism 3730 automated DNA sequencer by Shanghai Majorbio Bio-pharm Technology Co., Ltd and Beijing Genomics Institute. All sequences have been deposited in GenBank (Table 1).

### Phylogenetic analyses

DNA sequences were aligned in MEGA 6 (Tamura et al. 2013) by the Clustal W algorithm with default parameters (Thompson et al. 1997). Two gene segments, 535 base pairs (bp) of 16S and 645 bp of CO1, were concatenated seriatim into a 1180-bp single sequence. The dataset was partitioned according to the genes and codon positions, and then tested respectively in jmodeltest v2.1.2 with Akaike and Bayesian information criteria, all resulting in the best-fitting nucleotide substitution models of GTR + I + G. Sequenced data was analyzed using Bayesian inference (BI) in MrBayes 3.2.4 (Ronquist et al. 2012). Three independent runs were conducted in BI analysis, each of which was performed for 2,000,000 generations and sampled every 1000 generations with the first 25% samples were discarded as burn-in, resulting a potential scale reduction factor (PSRF) of < 0.01. Pairwise distances (p-distance) were calculated in MEGA 6 using the uncorrected p-distance model.

### Morphometrics

All specimens were fixed in 10 % buffered formalin and later transferred to 70% ethanol for preservation, and deposited at the Museum of Biology, Sun Yat-sen University (**SYS**) and Chengdu Institute of Biology, the Chinese Academy of Sciences (**CIB**), China.

Measurements follow Fei et al. (2009), and were taken with digital calipers to the nearest 0.1 mm. These measurements were as follows:

**SVL** snout‒vent length (from tip of snout to vent);

**HDL** head length (from tip of snout to rear of jaws);

**HDW** head width (head width at commissure of jaws);

**SNT** snout length (from tip of snout to anterior corner of eye);

**ED** eye diameter (diameter of exposed portion of eyeball);

**IOD** interorbital distance (minimum distance between upper eyelids);

**IND** internasal distance (distance between nares);

**TD** tympanum diameter (horizontal diameter of tympanum);

**TED** tympanum–eye distance (distance from anterior edge of tympanum to posterior corner of eye);

**HND** hand length (distance from distal end of radioulna to tip of phalanx of finger III);

**RAD** radioulna length;

**TIB** tibia length (distance from knee to heel);

**FTL** foot length (distance from distal end of tibia to tip of distal phalanx of toe IV).

Sex was determined by direct observation of calls, the presence of internal vocal sac openings and the presence of testicles observed through dissection for males, as well as the presence of eggs and ovaries on the abdomen through anatomise for females. Presence or absence of nuptial pads/spines was examined with a microscope.

Comparative morphological data of Megophrys species allocated to the subgenus Panophrys (currently contains 32 species) (Mahony et al. 2017; Tapley et al. 2017; Wang et al. 2017a; Wang et al. 2017b; Zhang et al. 2017; Li et al. 2018; Tapley et al. 2018), and a small-sized species *M.feii* (incertae sedis), were obtained from examination of museum specimens (see Appendix 1) and from the literature (Table 2). The order of the new species accounts follows the distributions of the new species that located in the longitudinal mountain belt from the south to the north.

**Table 2.** References for morphological characters for congeners of the subgenus Panophrys and *Megophrysfeii* (incertae sedis).

ID	Subgenus Panophrys	Literature obtained
1	*M.acuta* Wang, Li & Jin, 2014	Li et al. 2014
2	*M.baolongensis* Ye, Fei & Xie, 2007	Ye et al. 2007
3	*M.binchuanensis* Ye & Fei, 1995	Ye and Fei 1995
4	*M.binlingensis* Jiang, Fei & Ye, 2009	Fei et al. 2009
5	*M.boettgeri* (Boulenger, 1899)	Fei et al. 2012
6	*M.brachykolos* Inger & Romer, 1961	Inger and Romer 1961
7	*M.caudoprocta* Shen, 1994	Fei et al. 2012
8	*M.cheni* (Wang & Liu, 2014)	Wang et al. 2014
9	*M.daweimontis* Rao & Yang, 1997	Fei et al. 2012
10	*M.fansipanensis* Tapley, Cutajar, Mahony, Nguyen, Dau, Luong, Le, Nguyen, Nguyen, Portway, Luong & Rowley, 2018	Tapley et al. 2018
11	*M.huangshanensis* Fei & Ye, 2005	Fei et al. 2012
12	*M.hoanglienensis* Tapley, Cutajar, Mahony, Nguyen, Dau, Luong, Le, Nguyen, Nguyen, Portway, Luong &, 2018	Tapley et al. 2018
13	*M.insularis* (Wang, Liu, Lyu, Zeng & Wang, 2017)	Wang et al. 2017b
14	*M.jingdongensis* Fei & Ye, 1983	Fei et al. 2012
15	*M.jinggangensis* (Wang, 2012)	Wang et al. 2012
16	*M.kuatunensis* Pope, 1929	Fei et al. 2012
17	*M.latidactyla* Orlov, Poyarkov & Nguyen, 2015	Orlov et al. 2015
18	*M.leishanensis* Li, Xu, Liu, Jiang, Wei & Wang, 2018	Li et al. 2018
19	*M.liboensis* (Zhang, Li, Xiao, Li, Pan, Wang, Zhang & Zhou, 2017)	Zhang et al. 2017
20	*M.lini* (Wang &Yang, 2014)	Wang et al. 2014
21	*M.lishuiensis* (Wang, Liu & Jiang, 2017)	Wang et al. 2017a
22	*M.minor* Stejneger, 1926	Wang et al. 2017b
23	*M.obesa* Wang, Li & Zhao, 2014	Li et al. 2014
24	*M.ombrophila* Messenger & Dahn, 2019	Messenger et al. 2019
25	*M.omeimontis* Liu, 1950	Fei et al. 2009
26	*M.palpebralespinosa* Bourret, 1937	Fei et al. 2012
27	*M.rubrimera* Tapley, Cutajar, Mahony, Chung, Dau, Nguyen, Luong & Rowley, 2017	Tapley et al. 2017
28	*M.sangzhiensis* Jiang, Ye & Fei, 2008	Jiang et al. 2008
29	*M.shuichengensis* Tian & Sun, 1995	Tian et al. 2000
30	*M.spinata* Liu & Hu, 1973	Fei et al. 2012
31	*M.tuberogranulatus* Shen, Mo & Li, 2010	Mo et al. 2010
32	*M.wuliangshanensis* Ye & Fei, 1995	Ye and Fei 1995
33	*M.wushanensis* Ye & Fei, 1995	Ye and Fei 1995
**Incertae sedis**		
1	*M.feii* Yang, Wang & Wang, 2018	Yang et al. 2018

## Results

### Phylogenetics

**Figure 2.**
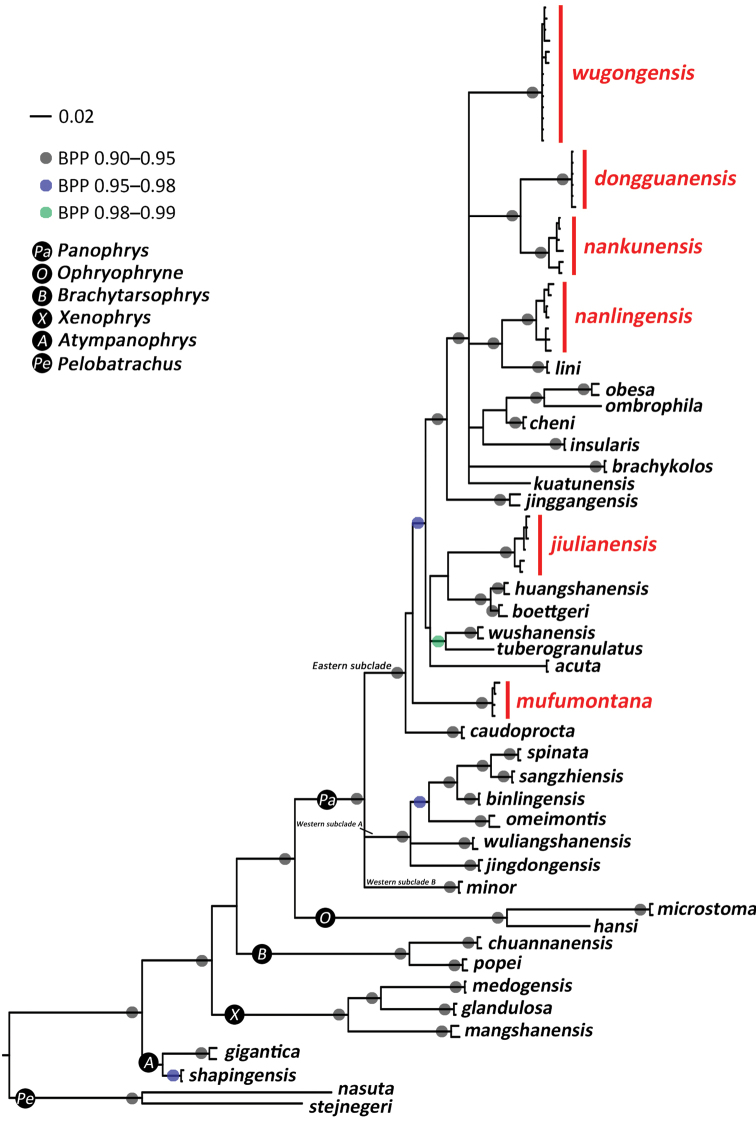
Bayesian inference tree derived from partial DNA sequences of the mitochondrial 16S rRNA + CO1 genes.

The Bayesian inference (BI) phylogenetic tree was integrated in Figure 2; the *p*-distances at the mitochondrial 16S rRNA gene fragment among all samples of the subgenus Panophrys were given in Table 3.

**Table 3.** Uncorrected *p*-distances among Megophrys species of the subgenus Panophrys in this study, based on mitochondrial 16S r RNA genes.

Species & ID No.	(1)–(6)	(7)–(12)	(13)–(16)	(17)–(22)	(23)–(29)	(30)–(42)	(43)–(44)	(45)–(46)	(47)–(48)	(49)–(50)	(51)–(52)	(53)–(54)	(55)–(56)	(57)–(58)													
*M.dongguanensis* sp. nov. (1)–(6)	0–0.2																										
*M.jiulianensis* sp. nov. (7)–(12)	5.3–5.8	0–0.7																									
*M.mufumontana* sp. nov. (13)–(16)	6.3	3.7–4	0																								
*M.nankunensis* sp. nov. (17)–(22)	2.6–2.8	4.7–4.9	4.9–5.1	0–0.7																							
*M.nanlingensis* sp. nov. (23)–(29)	5.3–6.1	5.3–6.3	5.8–6.5	4.9–5.8	0–0.7																						
*M.wugongensis* sp. nov. (30)–(42)	5.3–5.4	4–4.2	6–6.1	4.7–4.9	4.9–5.4	0																					
*M.acuta* (43)–(44)	7.4–7.5	7–7.4	7.9	7.9–8.1	6.7–7.2	8.1–8.2	0																				
*M.binlingensis* (45)–(46)	6.3	4.9–5.4	5.1	5.8–6.1	5.6–6.1	5.4	8.2	0																			
*M.boettgeri* (47)–(48)	5.1–5.4	2.3–2.8	2.8–3	4–4.4	4.4–5.4	4.2–4.4	6.5–6.8	4.2–4.4	0–0.2																		
*M.brachykolos* (49)–(50)	6.8	5.8–6.3	6.8	5.8–6.1	6.1–7.5	7.7	7.2	7.7	6.1	0–0.1																	
*M.caudoprocta* (51)–(52)	6.3	3.5–3.7	4	4.9–5.1	6.8–7.5	5.8	6.8	4.4	3.3	6.5	0																
*M.cheni* (53)–(54)	3–3.3	3.5–4	4.2–4.4	5.8–6	3.5–4.4	5.7–6.6	7–7.2	4.7–4.9	2.6	5.1–5.4	4.4–4.7	0–0.2															
*M.huangshanensis* (55)–(56)	5.6	3–3.3	3.7	4.7–4.9	5.1–5.8	4.7	6.5	4.9	0.9–1.2	6.5	3.5	3.3–3.5	0														
*M.insularis* (57)–(58)	4.4–4.9	5.1–5.8	5.1–6.5	3.5–4.2	4.4–5.8	4.9–5.1	7.7–8.4	5.8	4.4–5.1	6.3–6.5	6.1–6.5	3.3–3.5	4.7–5.4	0													
*M.jingdongensis* (59)–(60)	7.4	6–6.3	5.8	5.8–6	6.7–7.5	5.3–5.4	7.9	6.2–6.7	4.9–5.1	7.9	4.7	4.7–4.9	5.6	6.7	0												
*M.jinggangensis* (61)–(62)	5.8–6.3	4.4–4.9	4.2–4.4	4.7–5.1	4.9–5.8	4.4	6.7	4.9	3.7–4	6.1	5.4	3–3.3	4.4	4.7	5.3	0–0.5											
*M.kuatunensis* (63)	3.3	4–4.2	4.2	2.8–3	4.7–5.4	5.1	7.4	5.4	3.3–3.5	4.7	4.4	2.6–2.8	3.7	3.5	5.8	4.4	0										
*M.lini* (64)–(65)	5.1	5.1–5.6	6–6.1	4.7–4.9	3.7–4.4	5.6	6.5	6.3	4.2–4.4	4.9	5.8	3.3–3.5	4.7	4.9	6.3	4.4	3.7	0									
*M.minor* (66)–(67)	7.9–8.2	7–7.7	6.8–7	7.7–8.2	7–7.2	6.5–6.8	8.9–9.1	6.3–6.5	5.6–5.8	8.7–8.9	7–7.2	6.5–6.8	6.3–6.5	8.4–8.6	7.2–7.5	6.8–7	6.5–6.8	7.7–7.9	0–0.2								
*M.obesa* (68)–(69)	4.7–5.8	5.6–6	6.3	4.2	4.4–4.9	5.1	7.4	7.2	4.2–4.4	6.3	6.8	2.8–3	4.9–5.1	4.9	6.3	4.9–5.3	4.2	4.2	7.2–7.5	0							
*M.ombrophila* (70)	5.1	5.6–5.8	6–6.1	4.4–4.7	6–6.8	5.3–5.4	8.8	6.8	4.7–4.9	7	6.8	3–3.3	5.1–5.4	4.7	6.7	5.3–5.8	4.9	5.8	8.2–8.4	3	0						
*M.omeimontis* (71)–(72)	5.8	4.2–4.4	4.9	4.7–4.9	5.1–5.8	4.3–4.4	7.9	5.5	4–4.2	7	4.2	3.7–4	4.7	5.4	3	4.4–4.9	4.7	5.1	5.8–6.1	5.6	5.6	0					
*M.sangzhiensis* (73)–(74)	6.3	5.3–5.8	5.8	6.3	5.6–6	5.1	8.6	6.9	4.7–4.9	8.2	4.9	4.7–4.9	5.4	5.6	3.9	5.6	5.8	6.3	7.2–7.5	7.2	6.5	4	0				
*M.spinata* (75)–(76)	6.5	5.1–5.6	5.8	6.3–6.5	5.3–6.1	4.9	8.4	4.5	4.4–4.7	8.4	5.1	4.7–4.9	4.7	6.3	3.9	5.8	6	5.6	6.5–6.8	7	6.7	3	2	0			
*M.tuberogranulatus* (77)	4.7	2.6–2.8	3	3.3–3.5	4.7–5.1	4	6.5	7.5	3.4	5.4	3	2.1–2.3	2.3	4.2	4	3	3	4.7	5.8–6.1	4.7–5.8	4.4	3.7	3.7	3.7	0		
*M.wushanensis* (78)–(79)	4.9–5.4	3.5–4	5.8	4.2–4.7	5.6–6.3	4.9–5.1	7.2–7.5	7.5–7.6	3–3.5	5.8	4.2–4.4	3.3–3.7	3.7–4	3.7–4	5.4–5.6	3.7–4	3.7–4	5.6–5.8	7.2–7.5	5.4–5.8	4.7–4.9	3.7–4	5.1–5.4	5.1–5.4	2.1	0	
*M.wuliangshanensis* (80)–(81)	7.2	5.3–5.8	4	5.8–6	7–7.2	6–6.1	8.4	6.5–6.7	4.9–5.1	7.5	5.6	5.1–5.4	5.4–5.6	6.7	3.7	5.8	5.1	6.7	6.5–6.8	5.8	6.5	3.5	4.9	4.4	4.2	4.7–5.1	0–0.5

In our phylogenic tree, all sequences of the genus *Megophrys* grouped into six clades with strong node support values, which were consistent with the results from Mahony et al. (2017) and Liu et al. (2018), and corresponded to the six subgenera: *Panophrys*, *Ophryophryne*, *Xenophrys*, *Atympanophrys*, *Brachytarsophrys* and *Pelobatrachus*. The subgenus Panophrys is further divided into three subclades, named western subclade A, western subclade B and eastern subclade.

The western subclade A is composed of *Megophrysomeimontis*, *M.binglingensis*, *M.sangzhiensis*, *M.spinata*, *M.wuliangshanensis* and *M.jingdongensis*, and the western subclade B is composed of *M.minor*, all of which are distributed in southwestern China.

The eastern subclade contains 14 known species from southeastern China, i.e. *M.boettgeri*, *M.huangshanensis*, *M.kuatunensis*, *M.brachykolos*, *M.insularis*, *M.cheni*, *M.lini*, *M.jinggangensis*, *M.obesa*, *M.ombrophila*, *M.acuta*, *M.sangzhiensis*, *M.caudoprocta*, *M.tuberogranulatus* and *wushanensis*, and other six lineages made up of samples from the aforementioned longitudinal mountain belt in the middle of southeastern China with significant genetic differences (Table 3).

Among them, all samples from Mt. Mufu, Hunan (samples 13–16 in Table 1) clustered into a basal lineage of an eastern subclade with strong node supports and almost have no molecular differences; further, this population can be distinguished from all known species and other undescribed lineages by distinctive morphological characters and significant molecular differences with a lowest *p*-distance of 2.8%. Therefore, the population from Mt. Mufu represented a separately evolving lineage, and is described as a new species, Megophrys (Panophrys) mufumontana sp. nov., below.

All samples from Mt. Wugong, Jiangxi (samples 30–42 from Yangshimu Scenic Area and Wugongshan Scenic Area) clustered into a lineage with strong node supporting values and almost no genetic differences, which was defined as a species and recognized as *M.* sp12 by Liu et al. (2018); further, the population from Mt. Wugong can be distinguished from all known species and other undescribed lineages by distinctive morphological differences and significant molecular differences with a lowest *p*-distance of 4%. Therefore, the population from Mt. Wugong represented a separately evolving lineage and is described as a new species, Megophrys (Panophrys) wugongensis sp. nov., below.

All samples from Mt. Yinping, Guangdong (samples 1–6) clustered into a lineage with strong node supportg values and small genetic differences (highest *p*-distance 0.2%), which was defined as a species and recognized as *M.* sp11 by Liu et al. (2018); samples 17–22 from Mt. Nankun, Guangdong clustered into a lineage with strong node support values and small genetic differences (highest *p*-distance 0.7%), which was defined as a species and recognized as *M.* sp10 by Liu et al. (2018); these two populations are sister taxa to each other with significant genetic differences (*p*-distances 2.6–2.8%), and can be further distinguished from all known species and other undescribed lineages by distinctive morphological differences and significant molecular differences. Therefore, the populations from Mt. Yinping and Mt. Nankun represented two separately evolving lineages, and are described as new species, Megophrys (Panophrys) dongguanensis sp. nov. and Megophrys (Panophrys) nankunensis sp. nov., below.

Samples 7–10 from Mt. Jiulian, Jiangxi and samples 11–12 from Mt. Nankun, Guangdong clustered into a lineage with small genetic differences (highest *p*-distance 0.7%), which is a sister subclade to *M.boettgeri* and *M.huangshanensis* with large genetic differences (lowest p-distance 2.3%); therefore, these samples represented a separately evolving lineage, which was defined as a species and recognized as *M.* sp30 by Liu et al. (2018), and is described as a new species, Megophrys (Panophrys) jiulianensis sp. nov., below.

Samples 26–29 from Mt. Qiyun, Jiangxi were defined as a species and recognized as *M.* sp6 by Liu et al. (2018) and the samples 23–25 from Nanling Nature Reserve, Guangdong were defined as a species and recognized as *M.* sp7 by Liu et al. (2018). Although the populations from two locations are divided into two branches, the highest *p*-distance is only 0.7%. Moreover, there are no distinct morphological characters that can distinguish them from each other. Herein, we considered these two populations as one taxon, which is the sister taxon to *M.lini* with large genetic differences (*p*-distances 3.7–4.4%), representing a new species and described as, Megophrys (Panophrys) nanlingensis sp. nov., below.

## Taxonomic accounts

### Megophrys (Panophrys) dongguanensis

Taxon classificationAnimaliaAnuraMegophryidae

J. Wang & Y.Y. Wang sp. nov.

http://zoobank.org/94DBE153-5A7C-4820-8E27-E9BE41C3A764

Fig. 3 , Table 4

#### Holotype.

SYS a001973, adult male, collected by Run-Lin Li on 13 December 2012 from Mt. Yinping, Xiegang County (22°54'17.20"N, 114°13'23.88"E; 132 m a.s.l.), Dongguan City, Guangdong Province, China.

#### Paratypes (10 males).

SYS a002007, adult male, collected on 17 March 2013 by Run-Lin Li from Mt. Yinping, Qingxi County (22°53'26.21"N, 114°10'14.82"E; 277 m a.s.l.), Dongguan City, China; adult males, SYS a001971/CIB110006, SYS a001972, 1974–1975, collected on 12–13 December 2012, SYS a001492–1495, collected on 23 December 2012 by Run-Lin Li from the same locality as the holotype (100–300 m a.s.l.).

#### Diagnosis.

(1) Body size small to moderate, SVL 30.2–39.3 mm in 11 adult male specimens; (2) head width slightly larger than head length, HDW/HDL ratio 1.04–1.09; (3) snout pointed in dorsal view; (4) tympanum distinct, moderate-sized, TD/ED ratio 0.42–0.60; (5) strong vomerine ridge bearing vomerine teeth; (6) margin of tongue not notched behind; (7) hindlimbs short, heels not meeting, tibio-tarsal articulation reaching the region between tympanum and eye; (8) presence of subarticular tubercles and absence of lateral fringes on fingers, relative finger lengths II < I ≤ IV < III; (9) toes with rudiment of webbing at their bases and without lateral fringes, subarticular tubercles only present at the base of each toe; (10) numerous granules present on dorsal surface of body, several large tubercles present on surface of flanks; (11) presence of a barely visible reddish horn-like tubercle at the edge of the upper eyelid; (12) supratympanic fold distinct, whitish; (13) yellowish brown dorsally, with an incomplete dark triangular marking between eyes and usually an X-shaped marking on back of trunk; (14) ventral surface black brown, with white spots on posterior surface of abandon; (15) males with a single subgular vocal sac; (16) presence of nuptial pads with darker nuptial spines on dorsal surface of the first and second fingers in adult males during breeding season, respectively.

#### Comparisons.

Comparative data of *Megophrysdongguanensis* sp. nov. with *M.feii* and the 33 recognized members of Megophryss.l. allocated to thesubgenusPanophrys are listed in Table 5.

With significantly smaller body size, SVL 30.2–39.3 mm in males, *Megophrysdongguanensis* sp. nov. differs from the eight members with larger SVL values: *M.baolongensis* (42.0–45.0 mm in males), *M.binlingensis* (45.1–51.0 mm in males), *M.caudoprocta* (81.3 mm in male), *M.jingdongensis* (53.0–56.5 mm in males), *M.omeimontis* (56.0–59.5 mm in males), *M.sangzhiensis* (54.7 mm in single male), *M.spinata* (47.2–54.4 mm in males) and *M.shuichengensis* (102.0–118.3 mm in males).

*Megophrysdongguanensis* sp. nov. differs from 12 species occurring in eastern and southern China (*M.acuta*, *M.brachykolos*, *M.boettgeri*, *M.cheni*, *M.huangshanensis*, *M.insularis*, *M.jinggangensis*, *M.kuatunensis*, *M.lini*, *M.lishuiensis*, *M.obesa* and *M.ombrophila*) by the following combination of characters: presence of vomerine teeth (vs. absent in *M.acuta*, *M.boettgeri*, *M.brachykolos*, *M.cheni*, *M.huangshanensis*, *M.kuatunensis*, *M.lini*, *M.lishuiensis*, *M.obesa* and *M.ombrophila*), margin of tongue not notched posteriorly (vs. notched in *M.boettgeri*, *M.cheni*, *M.huangshanensis*, *M.insularis* and *M.kuatunensis*), absence of lateral fringes on toes (vs. presence of narrow lateral fringes on toes in *M.acuta*, *M.jinggangensis* and *M.kuatunensis*; presence of wide lateral fringes on toes in *M.boettgeri*, *M.cheni* and *M.lini*), toes with rudimentary webbing (vs. toes without webbing in *M.lishuiensis*, *M.kuatunensis* and *M.ombrophila*), hindlimbs short, with heels not meeting when the flexed hindlimbs are held at right angles to the body axis (vs. hindlimbs comparatively longer, with heels meeting or overlapping in *M.cheni*, *M.boettgeri*, *M.kuatunensis*, *M.jinggangensis* and *M.lini*), tibio-tarsal articulation reaching the region between tympanum and eye when hindlimb is stretched along the side of the body (vs. reaching forward to the shoulder in *M.brachykolos* and to the posterior edge of tympanum in *M.insularis*).

From the remaining 10 species occurring in China, *Megophrysdongguanensis* sp. nov. can be distinguished by the presence of vomerine teeth (vs. absent in *M.binchuanensis*, *M.leishanensis*, *M.minor*, *M.tuberogranulatus*, *M.wuliangshanensis* and *M.wushanensis*), by the unnotched tongue (vs. tongue notched in *M.daweimontis*, *M.liboensis*, *M.minor* and *M.rubrimera*), by the absence of lateral fringes on toes (vs. wide in *M.binchuanensis*, *M.liboensis*, *M.palpebralespinosa* and *M.wushanensis* (in males); narrow in *M.rubrimera*), by the rudimentary webbing on toes (vs. toes without webbing in *M.rubrimera* and *M.wuliangshanensis*; at least one-fourth webbed in *M.palpebralespinosa*), by the heels not meeting when the flexed hindlimbs are held at right angles to the body axis (vs. heels meeting in *M.binchuanensis* and *M.tuberogranulatus*; heels meeting or overlapping in *M.minor* and *M.wushanensis*; heels overlapping in *M.leishanensis*, *M.liboensis*, *M.palpebralespinosa* and *M.wuliangshanensis*).

*Megophrysdongguanensis* sp. nov. differs from the remaining species, *M.fansipanensis*, *M.hoanglienensis* and *M.latidactyla*, by the small horn-like tubercle at edge of upper eyelid (vs. slightly large in *M.latidactyla*), by the unnotched tongue (vs. tongue notched in *M.fansipanensis*, *M.hoanglienensis* and *M.latidactyla*), by the absence of lateral fringes on toes (vs. wide in *M.latidactyla*), by the presence of rudimentary webbing on toes (vs. webbing indistinct or absent in *M.fansipanensis* and *M.hoanglienensis*).

*Megophrysdongguanensis* sp. nov. further differs from *M.feii*, for which molecular data are lacking and cannot be allocated to any subgenus base on morphology only (Yang et al. 2018) by the larger body size, SVL 30.2–39.3 mm in males (VS. 24.3–25.1 mm in males), presence of nuptial pad with nuptial spines in males during breeding season (vs. absent), presence of vomerine teeth (vs. absent), unnotched tongue (vs. slightly notched), absence of lateral fringes on toes (vs. moderate or wide), heels not meeting when the flexed hindlimbs are held at right angles to the body axis (vs. heels overlapping).

#### Description of holotype.

Adult male. Body moderate-sized, SVL 38.0 mm; head width slightly larger than head length, HWD/HDL 1.09; snout pointed in dorsal view, projecting, sloping backward to mouth in profile, protruding well beyond margin of lower jaw; top of head flat; eye large, ED/HDL 0.40, pupil vertical; nostril oblique ovoid; canthus rostralis well developed, forming the beginning of a fleshy, protruding ridge, that continues over the upper eyelid, and transitions into a supratympanic fold that terminates in the scapular region; loreal region slightly oblique; internasal distance slightly larger than interorbital distance; tympanum distinct, moderate-sized, TD/ED 0.54; large ovoid choanae at the base of the maxilla; presence of vomerine ridge bearing vomerine teeth; margin of tongue not notched posteriorly; internal vocal slits present near the rear of the lower mandible.

Radioulna length and hand length 0.24 of SVL; fingers without webbing and lateral fringes, relative finger length II < I < IV < III; tips of fingers slightly dilated, round; presence of subarticular tubercles on finger III, and one subarticular tubercle at the bases of each finger; outer metacarpal tubercles indistinct, inner metacarpal tubercles distinct and observably enlarged. Hindlimbs short, tibio-tarsal articulation reaching the region between tympanum and eye when hindlimb is stretched along the side of the body; heels not meeting when the flexed hindlimbs are held at right angles to the body axis; tibia length 0.41 of SVL and foot length 0.61 of SVL; relative toe length I < II < V < III < IV; tips of toes round and slightly dilated; presence of rudimentary webbing on toes but absence of lateral fringes and tarsal folds; one subarticular tubercle at the bases of each toe; presence of a long ovoid inner metatarsal tubercle and absence of outer metatarsal tubercle.

Dorsal skin texture rough with dense granules; granules forming discontinuous X-shaped ridge with two discontinuous dorsolateral ridges on both sides at the central trunk; several large tubercles present on dorsal surface of flanks, thighs, shanks and forearms; four small tubercles present on the edge of upper eyelid, one of which is more prominent; distinct narrow supratympanic fold curving posteroventrally from posterior corner of eye to a level above insertion of arm; ventral skin texture smooth, several granules present on surface of abandon, ventral and posterior surface of thighs; pectoral gland small, closer to axilla; single femoral gland on rear of thigh.

#### Measurements of holotype (in mm).

SVL 38.0, HDL 12.0, HDW 13.1, SNT 4.5, IND 3.9, IOD 3.6, ED 4.8, TD 2.6, TED 2.1, HND 9.1, RAD 9.2, FTL 23.2, TIB 15.6.

#### Coloration of holotype in life.

**Figure 3.**
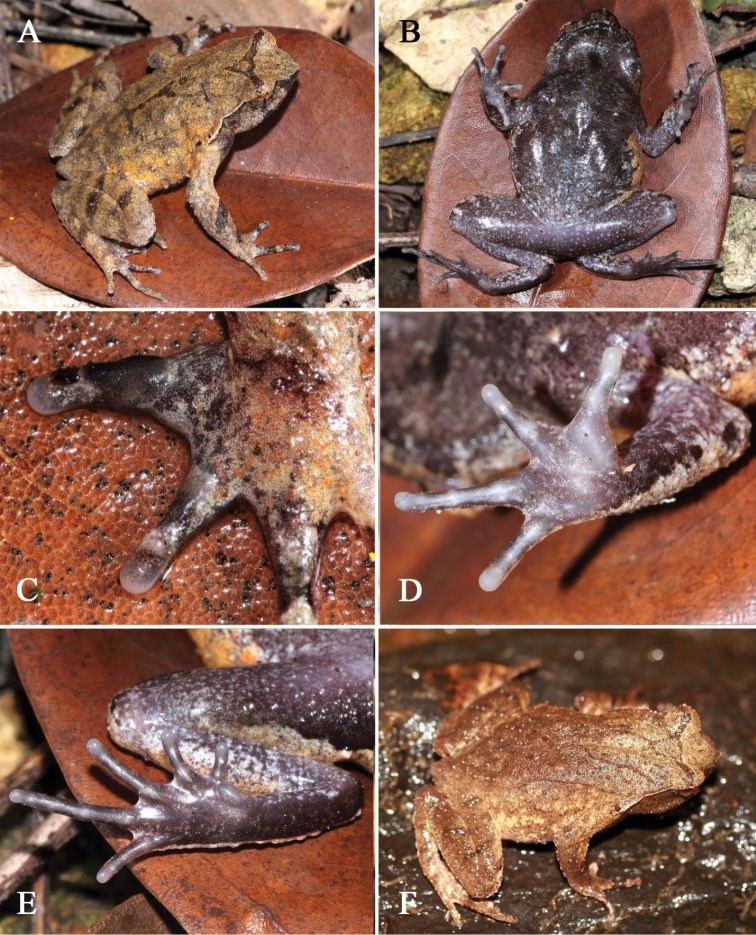
*Megophrysdongguanensis* sp. nov. in life: **A–E**SYS a001973, the male holotype **F**SYS a001492, a male paratype with more distinct skin ridges, granules and tubercles on dorsal surface of body.

(Fig. 3A–E) Yellowish brown dorsally, with a dark triangular marking between eyes. A wide oblique black band present on forearm. Dorsal surface of fingers and hindlimbs with dark grey transverse bands. Point of snout dark brown, presence of a vertical dark brown band below the eye. Tubercles on the edge of upper eyelid reddish. Supratympanic fold whitish tan. Ventral surface dark brown, with a black longitudinal band on surface of throat, several white spots present on ventral surface of limbs. Digits, inner and outer metacarpal tubercles greyish white, inner metatarsal tubercle greyish brown. Pectoral glands and femoral glands white. Iris yellowish brown.

#### Coloration of holotype in preservative.

Yellowish brown fades to greyish brown dorsally. Triangular marking between eyes, oblique bands on dorsal forearms, transverse bands on dorsal fingers and hindlimbs become indistinct. Color of ventral surface fades, all bands and spots become indistinct.

#### Variation.

Measurements of type series are listed in Table 4. All paratypes are very similar to holotype in morphology and color pattern. However, one male (SYS a001492) has more distinct skin ridges, granules and tubercles on dorsal surface of body (Fig. 3, F).

**Table 4.** Measurements (in mm; minimum-maximum, mean ± SD) of the type series of *Megophrysdongguanensis* sp. nov. and *M.nankunensis*. sp. nov., respectively.

Species	*Megophrysdongguanensis* sp. nov.	*Megophrysnankunensis* sp. nov.	
	Males (n = 9)	Males (n = 11)	Females (n = 2)
SVL	30.2–39.3 (36.3 ± 3.3)	29.9–34.9 (32.7 ± 1.5)	39.4–41.9
HDL	11.1–12.6 (12.0 ± 0.5)	9.0–11.3 (10.0 ± 0.7)	11.9–12.5
HDW	11.5–13.2 (12.8 ± 0.6)	10.1–12.6 (10.9 ± 0.7)	13.0–13.7
SNT	3.7–4.6 (4.2 ± 0.3)	2.8–3.7 (3.3 ± 0.3)	4.1–4.4
IND	3.7–3.9 (3.6 ± 0.2)	2.2–3.8 (3.1 ± 0.5)	3.1–3.9
IOD	3.2–3.6 (3.5 ± 0.2)	2.4–3.4 (2.8 ± 0.3)	3.1–3.2
ED	4.6–5.2 (4.9 ± 0.2)	3.1–4.4 (3.8 ± 0.4)	4.7–5.2
TD	2.1–2.8 (2.5 ± 0.3)	1.4–2.4 (1.9 ± 0.3)	2.6–2.7
TED	1.9–2.4 (2.1 ± 0.1)	0.8–1.7 (1.2 ± 0.2)	2.2–2.3
HND	8.3–9.8 (9.2 ± 0.5)	6.7–8.6 (7.5 ± 0.5)	9.7–10.2
RAD	8.2–9.7 (9.2 ± 0.5)	5.5–8.4 (6.6 ± 0.7)	8.0–8.4
FTL	13.4–16.5 (15.5 ± 1.08)	10.9–14.1 (12.2 ± 0.7)	14.6–15.3
TIB	19.9–23.4 (22.2 ± 1.11)	16.3–21.6 (18.2 ± 1.4)	22.6–25.5
HDL/SVL	0.32–0.37 (0.33 ± 0.02)	0.27–0.33 (0.31 ± 0.02)	0.30
HDW/SVL	0.33–0.40 (0.35 ± 0.02)	0.30–0.37 (0.33 ± 0.02)	0.33
HDW/HDL	1.04–1.09 (1.06 ± 0.02)	1.00–1.20 (1.10 ± 0.06)	1.09–1.10
SNT/HDL	0.33–0.37 (0.35 ± 0.01)	0.28–0.40 (0.33 ± 0.03)	0.35
SNT/SVL	0.11–0.12 (0.12 ± 0.01)	0.08–0.11 (0.10 ± 0.01)	0.10–0.11
IND/HDW	0.26–0.30 (0.28 ± 0.01)	0.21–0.34 (0.28 ± 0.04)	0.24–0.28
IOD/HDW	0.27–0.28 (0.27 ± 0.01)	0.23–0.30 (0.26 ± 0.02)	0.23–0.24
ED/HDL	0.37–0.44 (0.40 ± 0.02)	0.34–0.41 (0.38 ± 0.02)	0.39–0.42
ED/SVL	0.12–0.16 (0.14 ± 0.02)	0.09–0.13 (0.11 ± 0.01)	0.12
TD/ED	0.42–0.60 (0.51 ± 0.06)	0.43–0.61 (0.50 ± 0.06)	0.49–0.57
TED/TD	0.73–1.09 (0.88 ± 0.14)	0.58–0.74 (0.66 ± 0.05)	0.85
HND/SVL	0.24–0.28 (0.25 ± 0.01)	0.21–0.25 (0.23 ± 0.01)	0.24–0.25
RAD/SVL	0.24–0.28 (0.25 ± 0.01)	0.16–0.25 (0.20 ± 0.02)	0.20
TIB/SVL	0.41–0.46 (0.43 ± 0.02)	0.35–0.42 (0.37 ± 0.01)	0.37
FTL/SVL	0.58–0.70 (0.61 ± 0.04)	0.53–0.62 (0.56 ± 0.03)	0.57–0.61

**Table 5.** Diagnostic characters separating the seven new species described in this study from *Megophrysfeii* (incertae sedis) and 33 recognizing species of the Megophryss.l. allocated to thesubgenusPanophrys.

Species	SVL		Horn-like tubercle at edge of upper eyelid^1^	Vomerine teeth^2^	Tongue^3^	Lateral fringes on toes^4^	Toes^5^	TD/ED	TIB/SVL
	males (N)	females (N)							
*** M. dongguanensis ***	30.2–39.3 (9)	/	+	+	‒	‒	+	0.42–0.60	0.41–0.46
*** M. nankunensis ***	29.9–34.9 (11)	39.4–41.9 (2)	+	+	‒	‒	+	0.43–0.61	0.35–0.42
*** M. jiulianensis ***	30.4–33.9 (9)	34.1–37.5 (2)	+	+	+	‒	+	0.50–0.59	0.44–0.48
*** M. nanlingensis ***	30.5–37.3 (10)	/	+	+	+	+	+	0.43–0.57	0.45–0.51
*** M. wugongensis ***	31.0–34.1 (4)	38.5–42.8 (9)	+	‒	‒	‒	+	0.45–0.53	0.37–0.44
*** M. mufumontana ***	30.1–30.8 (2)	36.3 (2)	+	‒	‒	+	+	0.51–0.58	0.47–0.53
*M.acu*ta	27.1–33.0 (10)	28.1–33.6 (4)	++	‒	‒	+	+	0.57–0.71	0.38–0.45
* M. baolongensis *	42.0–45.0 (5)	/	+	‒	+	‒	‒	0.41	0.46
* M. binchuanensis *	32.0–36.0 (4)	40.2–42.5 (2)	‒	‒	+ or ‒	++	+	0.33–0.50	0.46–0.48
* M. binlingensis *	45.1–51.0 (3)	/	‒	‒	+	/	+	0.47–0.52	0.52–0.53
*M.boettger*i	34.5–37.8 (20)	39.7–46.8 (10)	+	‒	+	++	+	0.40–0.67	0.45–0.49
* M. brachykolos *	33.7–39.3 (5)	33.9–45.9 (2)	+	‒	‒	‒	+	> 0.50	0.37–0.42
* M. caudoprocta *	81.3 (1)	/	++	+	‒	/	+	0.50	0.51
* M. cheni *	26.2–29.5 (15)	31.8–34.1 (3)	+	‒	++	++	+	0.41–0.54	0.50–0.54
* M. daweimontis *	34.0–37.0 (18)	40.0–46.0 (3)	+	+	/	‒	‒	/	0.54
* M. fansipanensis *	30.9–44.3 (13)	41.7–42.5 (2)	+	+	+	‒	‒	0.53–0.80	0.49–0.59
* M. feii *	24.3–25.1 (4)	28.2–28.9 (2)	+	‒	+	++	+	0.51–0.58	0.48–0.55
* M. hoanglienensis *	37.4–47.6 (11)	59.6 (1)	+	+	+	‒	‒	0.54–0.75	0.44–0.63
*M.huangshanensi*s	36.0–41.6 (4)	44.2 (1)	+	‒	+	‒	‒	<0.50	0.42–0.45
* M. insularis *	36.8–41.2 (5)	47.1 (1)	+	+	+	‒	+	0.46–0.57	0.40–0.43
* M. jingdongensis *	53.0–56.5 (3)	63.5 (1)	+	+	+	++	+++	/	0.58–0.59
* M. jinggangensis *	35.1–36.7 (2)	38.4–41.6 (3)	++	+	‒	+	+	0.73–0.88	0.47–0.50
* M. kuatunensis *	26.2–29.6 (13)	37.4 (1)	+	‒	+	+	‒	0.44	0.38–0.48
* M. latidactyla *	38.9 (1)	/	++	+	‒	++	+	0.85	0.52
* M. leishanensis *	30.4–38.7 (10)	42.3 (2)	+	‒	‒	‒	+	/	/
* M. liboensis *	34.7–67.7 (5)	60.8–70.6 (8)	+++	+	+	++	+	0.48–0.78	0.44–0.61
* M. lini *	34.1–39.7 (20)	37.0–39.9 (4)	+	‒	‒	++	+	0.40–0.60	0.46–0.53
* M. lishuiensis *	30.7–34.7 (13)	36.9–40.4 (3)	+	‒	‒	‒	‒	/	/
*M.mino*r	34.5–41.2 (4)	/	‒	‒	+	‒	+	0.8–0.83	0.46–0.48
* M. obesa *	35.6 (1)	37.5–41.2 (6)	+	‒	‒	‒	+	0.51–0.66	0.41–0.47
* M. ombrophila *	27.4–34.5 (5)	32.8–35.0 (4)	+	‒	‒	‒	‒	0.52–0.69	0.32–0.41
* M. omeimontis *	56.0–59.5 (10)	68.0–72.5 (3)	+	+	+	+	+	/	0.52–0.56
* M. palpebralespinosa *	36.2–38.0 (2)	/	++	+	‒	++	+++	/	0.55
* M. rubrimera *	26.7–30.5 (8)	/	+	+	+	+	‒	0.58–0.76	0.48–0.56
* M. sangzhiensis *	54.7 (1)	/	+	+	+	+	+	0.62	0.59
* M. shuichengensis *	102.0–118.3 (7)	99.8–115.6 (6)	++	‒	+	++	+++	0.67	0.43–0.47
* M. spinata *	47.2–54.4 (18)	54.0–55.0 (2)	‒	‒	+	++	+++	0.43	0.56–0.58
* M. tuberogranulatus *	33.2–39.6 (9)	50.5 (1)	+ or ‒	‒	‒	‒	+	0.50	0.45–0.51
* M. wuliangshanensis *	27.3–31.6 (10)	41.0–41.5 (2)	‒	‒	+ or ‒	‒	‒	0.50	0.50–0.51
* M. wushanensis *	30.4–35.5 (10)	38.4 (1)	‒	‒	‒	‒ (in female), ++ (in male)	+	0.50	0.47–0.48

^1^ long point (+++); slightly large (++), small (+), absent or indistinct (‒); ^2^ present (+), or absent (‒); ^3^ notched (++), feebly notched (+), or not notched (‒); ^4^ wide (++), narrow (+), lacking (‒); ^5^ at least one-fourth webbed (+++), at most one-fourth webbed (++), with rudimentary webbing (+), or without webbing (‒).

#### Etymology.

The specific epithet “dongguanensis” is in reference to the type locality, Dongguan City of the new species. We propose the common English name “Dongguan Horned Toad” and Chinese name “Dong Guan Jiao Chan (东莞角蟾)”.

#### Distribution and natural history.

Currently, *Megophrysdongguanensis* sp. nov. is only known from Mt. Yinping, Guangdong Province, China. It inhabits flowing montane streams and the nearby forest floor and leaf litter at elevations between 100–300 m. Advertisement calls of males were noticed from mid-December until April of the next year just before the rainy season. Males were found calling on rocks in the flowing streams. Tadpoles could be found in this period.

### Megophrys (Panophrys) nankunensis

Taxon classificationAnimaliaAnuraMegophryidae

J. Wang, Zeng & Y.Y. Wang sp. nov.

http://zoobank.org/1F85DDB8-298D-47BB-B5BD-CA6D5A3E66A8

Fig. 4 , Table 4

#### Holotype.

SYS a004498, adult male, collected by Jian Wang and Hai-Long He on 20 October 2015 from Mt. Nankun (23°38'19"N, 113°53'24"E; 400 m a.s.l.), Longmen County, Huizhou City, Guangdong Province, China.

#### Paratypes (10 males & two females).

Adult females, SYS a004506–4507, collected by Jian Wang and Hai-Long He on 20 October 2015; adult males, SYS a002023, 2032–2033, collected by Run-Lin Li on 20 March 2013, SYS a004499–4504, SYS a004505/CIB110007, collected by Jian Wang and Hai-Long He on 20 October 2015, all from Mt. Nankun at elevations between 300–650 m.

#### Diagnosis.

(1) Body size small, SVL 29.9–34.9 mm in 11 adult males, 39.4–41.9 mm in two adult females; (2) head width slightly larger than head length, HDW/HDL ratio 1.00–1.20; (3) snout rounded in dorsal view, tip of snout slightly sharpened; (4) tympanum distinct, moderate-sized, TD/ED ratio 0.43–0.61; (5) strong vomerine ridge bearing vomerine teeth; (6) margin of tongue not notched behind; (7) shanks short, heels not meeting when the flexed hindlimbs are held at right angles to the body axis; tibia-tarsal articulation reaching forward to the region between tympanum and eye when hindlimb is stretched along the side of the body; (8) TIB/SVL ratio 0.35–0.42, FTL/SVL ratio 0.53–0.62; (9) absence of lateral fringes on fingers, presence of an indistinct subarticular tubercle on the bases of each finger, relative finger lengths II < I < IV < III; (10) toes with rudimentary webbing at their bases and without lateral fringes, subarticular tubercles only present on the bases of each toes; (11) dorsal surface with dense granules, surface of flanks and dorsal surface of limbs with large tubercles; (12) edge of eye lid with a small reddish horn-like tubercle; (13) supratympanic fold distinct, forming a depressed supraaxillary gland above insertion of arm; (14) dorsum beige to dark brown, with indistinct light brown patches, with an incomplete dark triangular marking between eyes; (15) males with a single subgular vocal sac, and dense dark villiform nuptial spines present on dorsal surface of first and second fingers during breeding season, respectively; (16) gravid females bear creamy yellow oocytes.

#### Comparisons.

Comparative data of *Megophrysnankunensis* sp. nov. with *M.dongduanensis* sp. nov., *M.feii* and the 33 recognized members of Megophryss.l. allocated to thesubgenusPanophrys are listed in Table 5.

In the ML and BI phylogenetic trees (Fig. 2), *Megophrysnankunensis* sp. nov. is a sister taxon to *M.dongguanensis* sp. nov. (*p*=4.6–5.0%) with high node-supporting value (0.1 in BI, 100% in ML%), and differs from the later by the snout rounded in dorsal view, tip of snout slightly sharpened (vs. snout pointed in dorsal view, tip of snout not sharpened), supratympanic fold forming a depressed supraaxillary gland above insertion of arm (vs. supraaxillary gland absent).

With significantly smaller body size, SVL 29.9–34.9 mm in males and 39.4–41.9 mm in females, *Megophrysnankunensis* sp. nov. differs from the 12 members with larger SVL values: *M.baolongensis* (42.0–45.0 mm in males), *M.binlingensis* (45.1–51.0 mm in males), *M.caudoprocta* (81.3 mm in single male), *M.hoanglienensis* (37.4–47.6 mm in males), *M.jingdongensis* (53.0–56.5 mm in males, 63.5 mm in single female), *M.latidactyla* (38.9 mm in single male), *M.omeimontis* (56.0–59.5 mm in males, 68.0–72.5 mm in females), *M.palpebralespinosa* (36.2–38.0 mm in males), *M.sangzhiensis* (54.7 mm in single male), *M.shuichengensis* (102.0–118.3 mm in males, 99.8–115.6 mm in females), *M.spinata* (47.2–54.4 mm in males, 54.0–55.0 mm in females) and *M.tuberogranulatus* (50.5 in single female).

*Megophrysnankunensis* sp. nov. differs from 12 species occurring in eastern and southern China (*M.acuta*, *M.brachykolos*, *M.boettgeri*, *M.cheni*, *M.huangshanensis*, *M.insularis, M.jinggangensis*, *M.kuatunensis*, *M.lini*, *M.lishuiensis*, *M.obesa* and *M.ombrophila*) by the following combination of characters: presence of vomerine teeth (vs. absent in *M.acuta*, *M.boettgeri*, *M.brachykolos*, *M.cheni*, *M.huangshanensis*, *M.kuatunensis*, *M.lini*, *M.lishuiensis*, *M.obesa* and *M.ombrophila*), absence of lateral fringes on toes (vs. presence of narrow lateral fringes on toes in *M.acuta*, *M.jinggangensis* and *M.kuatunensis*; presence of wide lateral fringes on toes in *M.boettgeri*, *M.cheni* and *M.lini*), toes with rudimentary webbing (vs. toes without webbing in *M.lishuiensis*, *M.kuatunensis* and *M.ombrophila*), hindlimbs short, with heels not meeting when the flexed hindlimbs are held at right angles to the body axis (vs. hindlimbs comparatively longer, with heels meeting or overlapping in *M.cheni*, *M.boettgeri*, *M.kuatunensis*, *M.jinggangensis* and *M.lini*), tibio-tarsal articulation reaching forward to the region between tympanum and eye when hindlimb is stretched along the side of the body (vs. reaching forward to the shoulder in *M.brachykolos* and to the posterior edge of tympanum in *M.insularis*), relative finger lengths II < I < IV < III (vs. IV < I < II < III in *M.brachykolos* and I < II < IV < III in *M.obesa* and *M.ombrophila*); supratympanic fold forming a depressed supraaxillary gland above insertion of arm (vs. supraaxillary gland swollen in *M.insularis*; absent in other 11 species).

*Megophrysnankunensis* sp. nov. differs from the remaining nine members of the *Megophryss.l.* allocated to the subgenus Panophrys which share a moderate or small body size, by the by the small horn-like tubercle at edge of upper eyelid (vs. horn-like tubercle indistinct or absent in *M.binchuanensis*, *M.minor*, *M.wuliangshanensis* and *M.wushanensis*; long point in *M.liboensis*), presence of vomerine teeth (vs. absent in *M.binchuanensis*, *M.leishanensis*, *M.minor*, *M.wuliangshanensis* and *M.wushanensis*), absence of lateral fringes on toes (vs. wide in *M.binchuanensis*, *M.liboensis*, *M.wushanensis* (wide in males); narrow in *M.rubrimera*), toes with rudimentary webbing (vs. toes without webbing in *M.daweimontis*, *M.rubrimera*, *M.wuliangshanensis* and *M.wushanensis* (in females); webbing indistinct or absent in *M.fansipanensis*), tibio-tarsal articulation reaching forward to the region between tympanum and eye when hindlimb is stretched along the side of the body (vs. reaching forward to the tip of snout in *M.daweimontis*), finger II shortest (vs. finger I shortest in *M.liboensis*), presence of an indistinct subarticular tubercle on the bases of each finger (vs. subarticular tubercle absent in *M.fansipanensis*), heels not meeting when the flexed hindlimbs are held at right angles to the body axis (heels meeting in *M.binchuanensis*; heels meeting or overlapping in *M.minor* and *M.wushanensis*; heels overlapping in *M.leishanensis*, *M.liboensis* and *M.wuliangshanensis*).

*Megophrysnankunensis* sp. nov. further differs from *M.feii*, for which molecular data are lacking and cannot be allocated to any subgenus base on morphology only (Yang et al. 2018) by the larger body size, SVL 29.9–34.9 mm in males and 39.4–41.9 mm in females (VS. 24.3–25.1 mm in males, 28.2–28.9 mm in females), presence of nuptial pad with nuptial spines in males during breeding season (vs. absent), presence of vomerine teeth (vs. absent), absence of lateral fringes on toes (vs. moderate or wide), heels not meeting when the flexed hindlimbs are held at right angles to the body axis (vs. heels overlapping).

#### Description of holotype.

Adult male. Habitus small, SVL 31.3 mm; head width slightly larger than head length, HDW/HDL 1.12; snout rounded in dorsal view, tip of snout slightly sharpened, sloping backward to mouth in profile, protruding well beyond margin of lower jaw; top of head flat; eye large, ED/HDL 0.38; nostril oblique ovoid; pupil vertical; canthus rostralis well developed, forming the beginning of a fleshy, protruding ridge, that continues over the upper eyelid, and transitions into a supratympanic fold that terminates in the scapular region; loreal region vertical; internasal distance slightly larger than interorbital distance; tympanum distinct, moderate-sized, TD/ED 0.44; large ovoid choanae at the base of the maxilla; strong vomerine ridge bearing vomerine teeth; margin of tongue weakly notched posteriorly; internal vocal slits present near the rear of the lower mandible.

RAD/SVL 0.22, HND/SVL 0.22; absence of lateral fringes and webbing on fingers, relative finger lengths II < I < IV < III; tip of finger rounded, slightly swollen; presence of a distinct subarticular tubercle on the base of each finger; outer metacarpal tubercles indistinct, inner metacarpal tubercles distinct and observably enlarged. Hindlimbs short, tibio-tarsal articulation reaching forward the anterior margin of tympanum when hindlimb is stretched along the side of the body; heels not meeting when the flexed hindlimbs are held at right angles to the body axis; TIB/SVL 0.37 and FTL/SVL 0.55; relative toe lengths I < II < V < III < IV; tips of toes round and slightly dilated; presence of rudimentary webbing on toes but absence of lateral fringes and tarsal folds; presence of a subarticular tubercle only at the bases of each toes; presence of a long ovoid inner metatarsal tubercle and absence of outer metatarsal tubercle.

Dorsal skin texture smooth with dense granules, some of which forming a weak X-shaped skin ridge on center of trunk; surface of flanks with large tubercles; presence of a small horn-like tubercle at the edge of eyelid; distinct supratympanic fold curving posteroventrally from posterior corner of eye to a level above insertion of arm, forming a swollen supraaxillary gland above insertion of arm; ventral skin texture smooth with granules on the surface of abdomen; pectoral gland large, equal size to tip of fingers, closer to axilla; single large femoral gland on rear of thigh.

#### Measurements of holotype (in mm).

SVL 31.3, HDL 9.6, HDW 10.8, SNT 3.4, IND 3.4, IOD 2.4, ED 3.7, TD 1.6, TED 1.0, HND 6.9, RAD 7.0, FTL 17.3, TIB 11.6.

#### Coloration of holotype in life.

**Figure 4.**
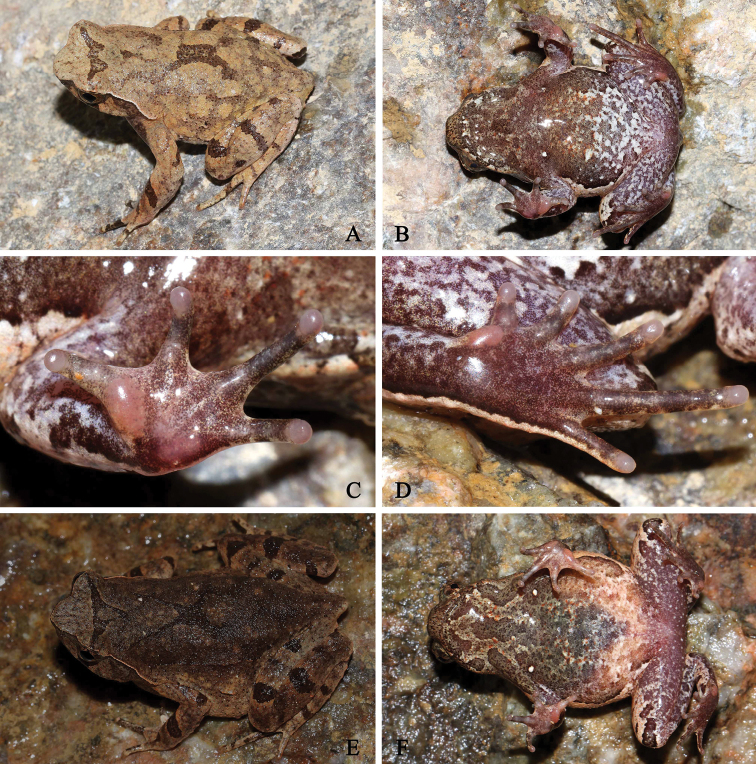
*Megophrysnankunensis* sp. nov. in life: **A–D**SYS a004498, the male holotype **E–F**SYS a004507, the female paratype.

(Fig. 4A–D) Dorsal surface beige with obscure darker patches, with a distinct and incomplete dark triangular marking between eyes, unconnected with an incomplete X-shaped marking on center of trunk. Forearm with dark bands dorsally; hindlimb with broad black transverse bands. Tip of snout dark brown. A dark brown vertical band below the eye. Supratympanic fold white. Horn-like tubercle at the edge of the upper eyelid orange. Surface of throat and chest dark brown, with scarlet spots. Posterior region of abdomen white, with dark brown and scarlet spots. Ventral surface of limbs white with brown patches. Ventral surface of hand and foot light brown, subarticular tubercle at the base of each fingers and toes, outer metacarpal tubercle, inner metatarsal tubercle and inner metacarpal tubercle pink. Pectoral and femoral glands white. Iris white.

#### Coloration of holotype in preservative.

On dorsal surface the beige fades to dark grey. Dark interorbital triangular marking becomes more indistinct. Ventral surface pale in color, grey-brownish grounding, markings and mottling more distinct, all scarlet spots absent.

#### Variation.

Measurements and body proportions of type series are given in Table 4.

All paratype specimens were very similar in morphology and color pattern. However, the holotype has the dorsal surface beige (vs. reddish brown in paratypes SYS a002033, 4501, and dark brown in paratypes SYS a004502–4506, 4507 (Fig. 4E–F)), dorsal skin texture smooth, granules and tubercles weak (vs. dorsal skin texture relatively rough with more distinct granules and tubercles in paratypes SYS a004502, 4504–4507), and ventral surface of hand and foot light brown (vs. ventral surface of hand and foot grey white in paratypes SYS a004502–4504).

#### Etymology.

The specific epithet “nankunensis” is in reference to the type locality of the new species: Mt. Nankun. We propose the common English name “Nankunshan Horned Toad” and Chinese name “Nan Kun Shan Jiao Chan (南昆山角蟾)”.

#### Distribution and habits.

**Figure 5.**
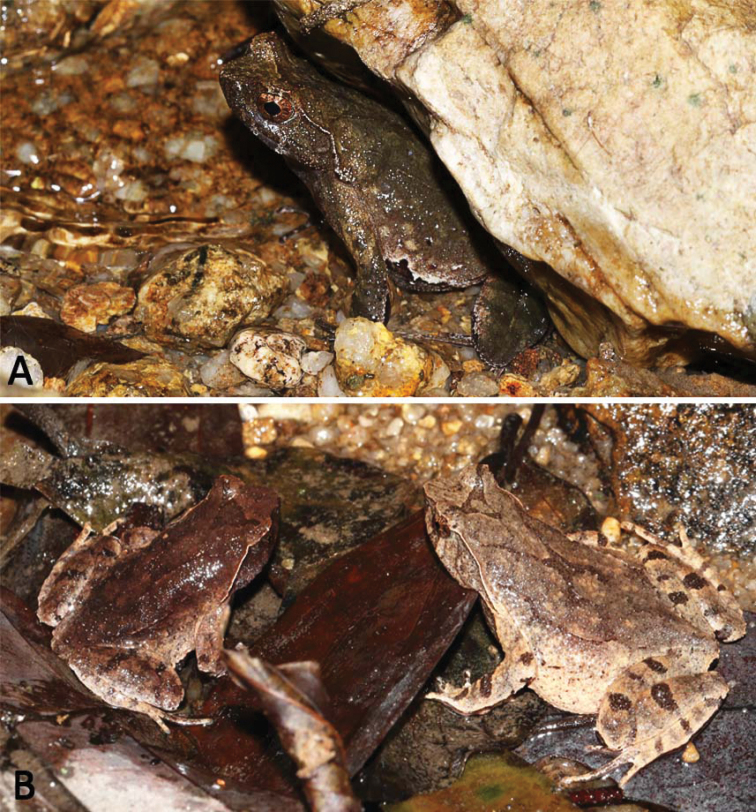
Ecology and behavior of *Megophrysnankunensis* sp. nov. **A** an adult male observed under the rock in the flowing stream **B** pair of *M.nankunensis* sp. nov. observed exposed on leave litters in a flowing stream, about 2.5 m wide, prior to amplexus.

Currently, *Megophrysnankunensis* sp. nov. is known only from the type locality, Mt. Nankun in Longmen County, Guangdong Province, China. It inhabits forest floor, leaf litter and the nearby undergrowth rocky mountainous streams (2–3 m wide) surrounded by moist subtropical evergreen broadleaved forests at elevations between 300–600 m. Breeding season of *M.nankunensis* sp. nov. is from October to the following March, males were found calling under the leaf litter or rocks (Fig. 5A) on the ground in the flowing streams, besides, a pair were observed exposed on the floor in a flowing stream, about 2.5 m wide, prior to amplexus (Fig. 5B) at 20:09 P.M. on 20 October 2015. Tadpoles were not observed in this period.

### Megophrys (Panophrys) jiulianensis

Taxon classificationAnimaliaAnuraMegophryidae

J. Wang, Zeng, Lyu & Y.Y. Wang sp. nov.

http://zoobank.org/2B18FD8D-520D-4531-AA9B-852E0E2AC92A

Fig. 6 , Table 6

#### Holotype.

SYS a002112, adult male, collected by Yu-Long Li on 2 May 2013 from Daqiutian Protection Station (24°34'34.99"N, 114°26'28.53"E; 560 m a.s.l.) of Mt. Jiulian, Longnan County, Ganzhou City, Jiangxi Province, China.

#### Paratypes (nine males & two females).

SYS a002110, 2111, adult females, collected by Yu-Long Li on 3 May 2013 from Xiagongtang Protection Station (24°32'16.74"N, 114°27'56.82"E; 770 m a.s.l.) of Mt. Jiulian; SYS a001007, 1009, adult males, collected by Run-Lin Li on 23 July 2010 from Daqiutian Protection Station of Mt. Jiulian; SYS a002107–2109, 2113–2114, SYS a002115/CIB110008, adult males, collected by Yu-Long Li on 1–4 May 2013 from Xiagongtang Protection Station and Daqiutian Protection Station of Mt. Jiulian at elevations between 400–800 m a.s.l.; SYS a002031, adult male, collected by Run-Lin Li on 20 Marth 2013 from Mt. Nankun (23°38'21.94"N, 113°50'39.49"E; 610 m a.s.l.), Longmen County, Huizhou City, Guangdong Province, China.

#### Diagnosis.

(1) Body slender and small-sized, SVL 30.4–33.9 mm in nine adult males, 34.1–37.5 mm in two adult females; (2) head width slightly larger than head length, HDW/HDL ratio 1.04–1.06; (3) snout rounded in dorsal view; (4) eye large, tympanum distinct, moderate-sized, TD/ED ratio 0.50–0.59; (5) weak vomerine ridge bearing vomerine teeth; (6) tongue weakly notched posteriorly; (7) hindlimbs slender, heels overlapping when the flexed hindlimbs are held at right angles to the body axis, tibia-tarsal articulation reaching forward to the middle of eye when hindlimb is stretched along the side of the body; (8) absence of lateral fringes on fingers, presence of an indistinct subarticular tubercle on the bases of each finger, relative finger lengths II < I < IV < III; (9) toes with rudimentary webbing at their bases and without lateral fringes, subarticular tubercles only present at the base of toe I and II; (10) dorsal skin rough, presence of black spines on granules of dorsal skin, and occasionally present on canthus rostralis and margin of tympanum, presence of large tubercles on flanks, dorsal body and limbs; (11) four prominent parallel dorsolateral ridges with granules bearing black spines on back of trunk, the middle two ridges forming a X-shaped ridge occasionally; (12) a reddish horn-like tubercle bearing a black spine at its tip at the edge of eye lid; (13) distinct supratympanic fold bearing black spines; (14) beige to brownish red above, with an hollow dark triangle between eyes and a rectangular dark marking on the center of the back of trunk; (15) males with a single subgular vocal sac, and presence of nuptial pads bearing darker nuptial spines on dorsal surface of the first and second fingers in adult males during breeding season, respectively; (16) gravid females bear creamy yellow oocytes.

#### Comparisons.

Comparative data of *Megophrysjiulianensis* sp. nov. with *M.dongduanensis* sp. nov., *M.nankunensis* sp. nov., *M.feii* and the 33 recognized members of the Megophryss.l. allocated to thesubgenusPanophrys are listed in Table 5.

*Megophrysjiulianensis* sp. nov. is sympatric with *M.nankunensis* sp. nov. in Mt. Nankun, but it can be easily distinguished from the later by heels overlapping when the flexed hindlimbs are held at right angles to the body axis (vs. heels not meeting), TIB/SVL ratio 0.61–0.68 (vs. TIB/SVL ratio 0.35–0.42), supratympanic fold not forming a supraaxillary gland above insertion of arm (vs. supratympanic fold forming a depressed supraaxillary gland), presence of black spines on dorsal skin (vs. absent); besides, *M.jiulianensis* sp. nov. differs from *M.dongguanensis* sp. nov. by the notched tongue vs. (not notched), heels overlapping when the flexed hindlimbs are held at right angles to the body axis (vs. heels not meeting), TIB/SVL ratio 0.61–0.68 (vs. TIB/SVL ratio 0.41–0.46).

With significantly smaller body size, SVL 30.4–33.9 mm in males and 34.1–37.5 mm in females, *M.jiulianensis* sp. nov. differs from the 17 members with larger SVL values: *M.baolongensis* (42.0–45.0 mm in males), *M.binchuanensis* (40.2–42.5 mm in females), *M.binlingensis* (45.1–51.0 mm in males), *M.caudoprocta* (81.3 mm in single male), *M.daweimontis* (40.0–46.0 mm in females), *M.fansipanensis* (41.7–42.5 mm in females), *M.hoanglienensis* (37.4–47.6 mm in males, 59.6 mm in single female), *M.jingdongensis* (53.0–56.5 mm in males, 63.5 mm in single female), *M.liboensis* (34.7–67.7 mm in males, 60.8–70.6 mm in females), *M.minor* (34.5–41.2 mm in males), *M.omeimontis* (56.0–59.5 mm in males, 68.0–72.5 mm in females), *M.palpebralespinosa* (36.2–38.0 mm in males), *M.sangzhiensis* (54.7 mm in single male), *M.shuichengensis* (102.0–118.3 mm in males, 99.8–115.6 mm in females), *M.spinata* (47.2–54.4 mm in males, 54.0–55.0 mm in females), *M.tuberogranulatus* (50.5 mm in single female) and *M.wuliangshanensis* (41.3 mm in single female).

*Megophrysjiulianensis* sp. nov. differs from 12 species occurring in eastern and southern China (*M.acuta*, *M.brachykolos*, *M.boettgeri*, *M.cheni*, *M.huangshanensis*, *M.insularis*, *M.jinggangensis*, *M.kuatunensis*, *M.lini*, *M.lishuiensis*, *M.obesa* and *M.ombrophila*) by the following combination of characters: presence of vomerine teeth (vs. absent in *M.acuta*, *M.boettgeri*, *M.brachykolos*, *M.cheni*, *M.huangshanensis*, *M.jinggangensis*, *M.kuatunensis*, *M.lini*, *M.lishuiensis*, *M.obesa* and *M.ombrophila*), tongue notched posteriorly (vs. not notched in *M.acuta*, *M.brachykolos*, *M.jinggangensis*, *M.lini*, *M.lishuiensis*, *M.obesa* and *M.ombrophila*), absence of lateral fringes on toes (vs. narrow in *M.acuta*, *M.jinggangensis* and *M.kuatunensis*; wide in *M.boettgeri*, *M.cheni* and *M.lini*), heels overlapping when the flexed hindlimbs are held at right angles to the body axis (vs. heels not meeting in *M.acuta*, *M.brachykolos*, *M.insularis, M.obesa* and *M.ombrophila*).

*Megophrysjiulianensis* sp. nov. differs from the remaining four members of the Megophryss.l. allocated to thesubgenusPanophrys which share a moderate or small body size, by the presence of vomerine teeth (vs. absent in *M.leishanensis* and *M.wushanensis*), tongue notched posteriorly (vs. not notched in *M.leishanensis*, *M.wushanensis* and *M.latidactyla*), absence of lateral fringes on toes (vs. narrow in *M.rubrimera*; wide in *M.latidactyla* and *M.wushanensis* (wide in females)), toe webbing rudimentary (vs. absence of webbing on toes in *M.rubrimera*).

*Megophrysjiulianensis* sp. nov. further differs from *M.feii*, for which molecular data are lacking and cannot be allocated to any subgenus base on morphology only (Yang et al. 2018) by the larger body size, SVL 30.4–33.9 mm in males and 34.1–37.5 mm in females (VS. 24.3–25.1 mm in males, 28.2–28.9 mm in females), presence of nuptial pad with nuptial spines in males during breeding season (vs. absent), presence of vomerine teeth (vs. absent), absence of lateral fringes on toes (vs. moderate or wide).

#### Description of holotype.

Adult male. Habitus slender and small, SVL 32.0 mm; head width slightly larger than head length, HDW/HWL 1.04; snout rounded in dorsal view, projecting, sloping backward to mouth in profile, protruding well beyond margin of lower jaw; top of head flat; eye large, ED/HDL 0.39; nostril oblique ovoid; pupil vertical; canthus rostralis well developed, forming the beginning of a fleshy, protruding ridge, that continues over the upper eyelid, and transitions into a supratympanic fold that terminates in the scapular region; loreal region vertical; internasal distance slightly larger than interorbital distance; tympanum distinct, moderate-sized, TD/ED 0.52; large ovoid choanae at the base of the maxilla; weak vomerine ridge bearing vomerine teeth; margin of tongue weakly notched posteriorly; internal vocal slits present near the rear of the lower mandible..

RAD/SVL 0.25; absence of lateral fringes and webbing on fingers, relative finger lengths II < I < IV < III; tip of finger rounded, slightly swollen; presence of an indistinct subarticular tubercle on the base of each finger; outer metacarpal tubercles indistinct, inner metacarpal tubercles distinct and observably enlarged. Hindlimbs long, tibio-tarsal articulation reaching forward to the middle of eye when hindlimb is stretched along the side of the body; heels overlapping when the flexed hindlimbs are held at right angles to the body axis; TIB/SVL 0.46 and FTL/SVL 0.62; relative toe lengths I < II < V < III < IV; tips of toes round and slightly dilated; presence of rudimentary webbing on toes but absence of lateral fringes and tarsal folds; presence of a subarticular tubercle only at the bases of the first and second toes; presence of a long ovoid inner metatarsal tubercle and absence of outer metatarsal tubercle.

Dorsum rough with dense granules bearing spines; canthus rostralis, margin of tympanum, supratympanic fold and upper lip with dense spines; presence of large tubercles bearing spines on dorsal surface of body, surface of flanks and dorsal and posterolateral surface of limbs; prominent parallel dorsolateral ridges with granules bearing spines on back of trunk; presence of a horn-like tubercle bearing a spine at its tip at the edge of eye lid; distinct supratympanic fold curving posteroventrally from posterior corner of eye to a level above insertion of arm; ventral skin texture smooth, the lower lip bears spines; sides of belly with large tubercles; ventral skin texture of thighs smooth with a few small tubercles, posterior surface and surface around anus with large tubercles bearing spines; surface of tibia-tarsal with a few tubercles bearing spines; presence of spines on lateral sides of fingers and toes; pectoral gland moderate-sized, closer to axilla; single femoral gland on rear of thigh, distinctly smaller than pectoral gland.

#### Measurements of holotype (in mm).

SVL 32.2, HDL 11.5, HDW 11.4, SNT 3.6, IND 3.5, IOD 3.3, ED 4.2, TD 2.3, TED 1.7, HND 8.0, RAD 8.1, FTL 20.5, TIB 14.7.

#### Coloration of holotype in life.

**Figure 6.**
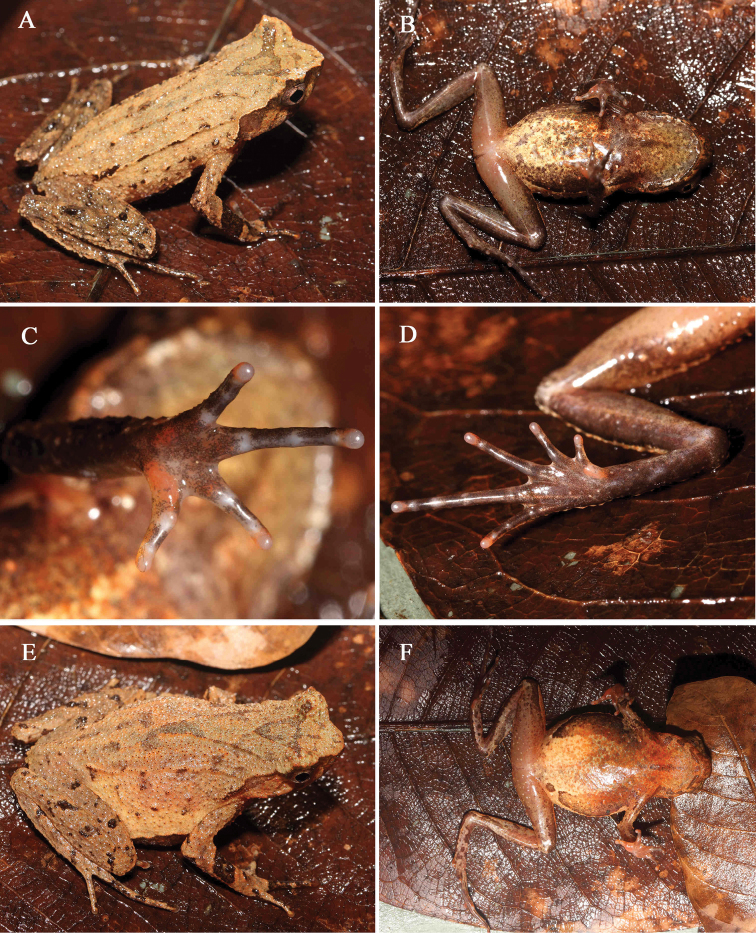
General life aspect in life of *Megophrysjiulianensis* sp. nov.: **A–D**SYS a002112, the male holotype **E–F**SYS a002111, the female paratype.

(Fig. 6A–D) Dorsal surface yellowish brown, with an incomplete dark triangular marking between eyes. Spines on dorsal surface, granules and tubercles black. Forearm with a distinct, black oblique band. Transverse bands on hindlimb indistinct. Tip of snout grayish brown. A grayish-brown vertical band below the eye. Tubercle at the edge of the upper eyelid red. Ventral surface yellow, scattered with dense dark gray spots and black scarlet blotches; ventral surface of limbs flesh colored with pink and black spots. Palms and soles dark brown, inner metatarsal tubercle, outer metacarpal tubercle and inner metacarpal tubercle orange red, tip of digits orange-red. Pectoral glands and femoral glands white. Iris white.

#### Coloration of holotype in preservative.

Dorsum yellowish brown fades to greyish brown, scattered with black spots. Greyish black triangular marking between the eyes become more distinct. Ventral surface paled in color, brown grounding, markings and mottling become more distinct.

#### Variation.

Measurements and body proportions of type series are given in Table 6.

All paratype specimens were very similar in morphology and color pattern. However, dorsal skin texture is more rough with well-developed spines in the female specimen SYS a002111 (Fig. 6E–F), dorsal surface yellowish brown in the other female specimen SYS a002110, and the middle two ridges on dorsum forming an X-shape skin ridge in the male specimen SYS a002108.

**Table 6.** Measurements (in mm; minimum-maximum, mean ± SD) of the type series of *Megophrysjiulianensis* sp. nov.

Species	*Megophrysjiulianensis* sp. nov.	
	Males (n = 9)	Females (n = 2)
SVL	30.4–33.9 (32.2 ± 1.2)	34.1–37.5
HDL	10.7–11.6 (11.2 ± 0.4)	12.0–12.4
HDW	10.9–11.8 (11.4 ± 0.4)	12.5–13.2
SNT	3.4–3.8 (3.6 ± 0.2)	3.9–4.1
IND	3.2–3.6 (3.5 ± 0.1)	3.5–3.8
IOD	3.2–3.5 (3.3 ± 0.1)	3.6
ED	3.9–4.4 (4.2 ± 0.2)	4.3–4.4
TD	2.1–2.5 (2.3 ± 0.1)	2.2–2.4
TED	1.6–2.0 (1.7 ± 0.1)	2.1–2.5
HND	7.4–10.6 (8.0 ± 0.4)	8.3–9.5
RAD	7.7–8.5 (8.1 ± 0.3)	8.3–9.8
FTL	14.1–15.2 (14.7 ± 0.4)	16.0–17.8
TIB	19.8–21.1 (20.5 ± 0.5)	21.6–25.5
HDL/SVL	0.34–0.37 (0.35 ± 0.01)	0.33–0.35
HDW/SVL	0.34–0.37 (0.35 ± 0.01)	0.35–0.37
HDW/HDL	1.00–1.04 (1.02 ± 0.02)	1.04–1.06
SNT/HDL	0.32–0.34 (0.32 ± 0.01)	0.33
SNT/SVL	0.11–0.12 (0.11 ± 0.00)	0.11
IND/HDW	0.29–0.33 (0.30 ± 0.01)	0.28–0.29
IOD/HDW	0.28–0.30 (0.29 ± 0.01)	0.27–0.29
ED/HDL	0.36–0.39 (0.38 ± 0.01)	0.35–0.36
ED/SVL	0.12–0.14 (0.13 ± 0.01)	0.12–0.13
TD/ED	0.50–0.59 (0.55 ± 0.03)	0.51–0.55
TED/TD	0.68–0.87 (0.75 ± 0.07)	0.95–1.04
HND/SVL	0.24–0.26 (0.25 ± 0.01)	0.24–0.25
RAD/SVL	0.24–0.27 (0.25 ± 0.01)	0.24–0.26
TIB/SVL	0.44–0.48 (0.46 ± 0.01)	0.47
FTL/SVL	0.61–0.67 (0.64 ± 0.02)	0.63–0.68

#### Etymology.

The specific epithet “jiulianensis” is in reference to the known localities of the new species: Mt. Jiulian and Nankunshan Natuire Reserve located in the Jiulian Mountains range. We propose the common English name “Jiulianshan Horned Toad” and Chinese name “Jiu Lian Shan Jiao Chan (九连山角蟾)”.

#### Distribution and natural history.

**Figure 7.**
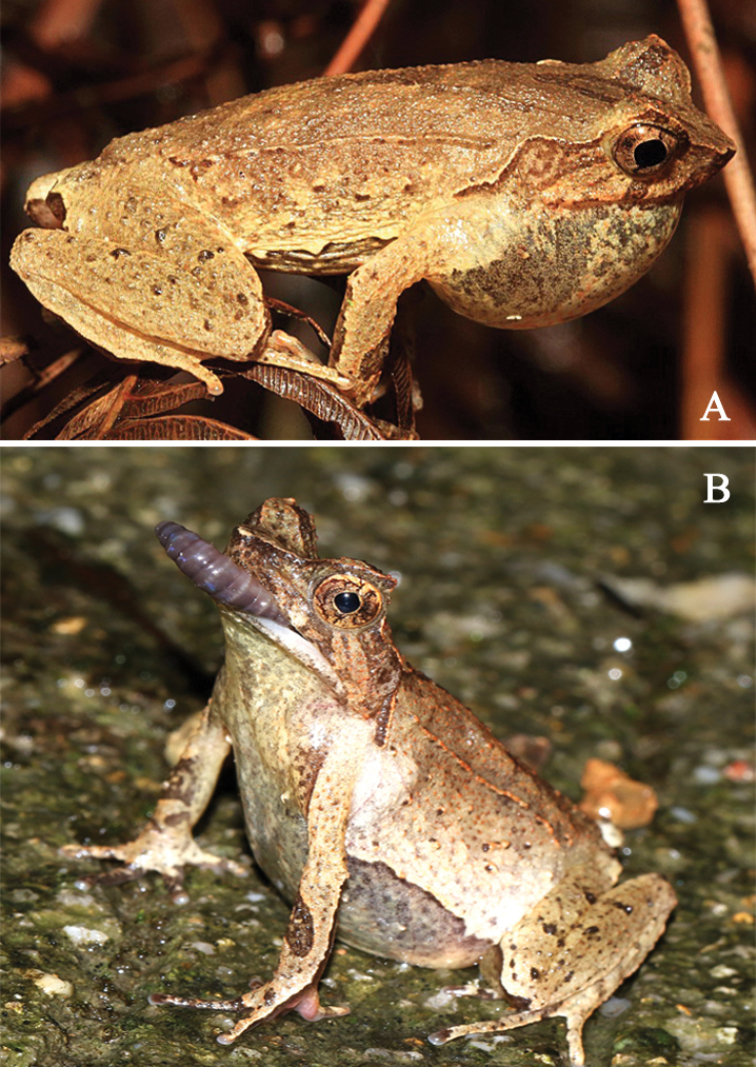
Ecology and behavior of *Megophrysjiulianensis* sp. nov.: **A** The male paratype SYS a002031 observed calling on a leaf (showing subgular vocal sac) **B** a female individual observed feeding on an earthworm after rain, both from Mt. Nankun in Guangdong Province.

Currently, *Megophrysjiulianensis* sp. nov. is known from Mt. Nankun in Guangdong Province and the type locality, Jiulian Nature Reserve in Jiangxi Province, China. It inhabits forest floor, leaf litter and the nearby undergrowth mountainous streams surrounded by moist subtropical evergreen broadleaved forests at elevations between 500–800 m. Breeding season of *M.jiulianensis* sp. nov. is from March to July, males were usually found staying while calling on leaves (Fig. 7A), about 0.1–0.3 m above the ground. After the rain, numerous individuals can be easily found on the road, and a female individual from Mt. Nankun was observed feeding on an earthworm (Fig. 7B) on 20:45 p.m., 21 March 2016. Tadpoles could be found all year round.

*Megophrysjiulianensis* sp. nov. is sympatric with *M.nankunensis* sp. nov. and *M.mangshanensis* at Mt. Nankun.

### Megophrys (Panophrys) nanlingensis

Taxon classificationAnimaliaAnuraMegophryidae

Lyu, J. Wang, Liu & Y.Y. Wang sp. nov.

http://zoobank.org/F9567F3F-D374-4CE8-A3A6-01C5F6A17A2D

Fig. 8 , Table 7

#### Holotype.

SYS a001964, adult male, collected by Run-Lin Li on 21 December 2012 from Nanling Nature Reserve (24°54'48.80"N, 113°01'12.34"E; 1008m a.s.l.), Ruyuan County, Shaoguan City, Guangdong Province, China.

#### Paratypes (nine males).

SYS a001959–1962, SYS a001963/CIB110010, adult males, collected on 21 December 2012 by Run-Lin Li from the same stream as the holotype (1000–1300 m a.s.l.); SYS a002334, 2356–2358, collected on 1–3 October 2013 by Ying-Yong Wang and Zu-Yao Liu from Mt. Qiyun (25°52'22.84"N, 114°01'52.09"E; 691–1355m a.s.l.), Chongyi County, Ganzhou City, Jiangxi Province, China.

#### Diagnosis.

(1) Body small-sized, SVL 30.5–37.3 mm in 10 adult males; (2) snout rounded in dorsal view; (3) tympanum distinct, moderate-sized, TD/ED ratio 0.43–0.57; (4) vomerine ridge and vomerine teeth present; (5) tongue notched posteriorly; (6) absence of lateral fringes and webbing on fingers, presence of narrow lateral fringes and rudimentary webbing on toes; (7) presence of a subarticular tubercle at the base of each finger and toe; (8) hindlimbs slender, heels overlapping, tibio-tarsal articulation reaching between the posterior corner to the center of eye; (9) TIB/SVL ratio 0.45–0.51 and FTL/SVL ratio 0.61–0.73; (10) dense conical granules present on surface of temporal region, upper lip, and from loreal region to the tip of snout; (11) granules and tubercles on dorsal surface forming a discontinuous X-shaped ridge and a pair of discontinuous dorsolateral ridges on back of trunk; (12) supratympanic fold distinct, whitish tan; (13) brown dorsally, with a dark triangular marking with light yellow edge between eyes, and an X-shaped or V-shaped marking with light yellow edge on the center of the back of trunk; (14) presence of a single subgular vocal sac in males; (15) nuptial pads and nuptial spines invisible in males during breeding season.

#### Comparisons.

Comparative data of *Megophrysnanlingensis* sp. nov. with *M.dongduanensis* sp. nov., *M.nankunensis* sp. nov., *M.jiulianensis* sp. nov., *M.feii* and the 33 recognized members of Megophryss.l. allocated to thesubgenusPanophrys are listed in Table 5.

*Megophrysnanlingensis* sp. nov. differs from *M.dongguanensis* sp. nov., *M.nankunensis* sp. nov. and *M.jiulianensis* sp. nov. by the heels overlapping when hindlimb is stretched along the side of the body (vs. heels not meeting in *M.dongguanensis* sp. nov. and *M.nankunensis* sp. nov.), presence of lateral fringes on toes (vs. absent in *M.dongguanensis* sp. nov., *M.nankunensis* sp. nov. and *M.jiulianensis* sp. nov.), tongue notched posteriorly (vs. not notched in *M.dongguanensis* sp. nov. and *M.nankunensis* sp. nov.), skin relatively smooth and lacking black horny spines (vs. skin rough with black horny spines in *M . jiulianensis* sp. nov.).

With the smaller body size, SVL 30.5–37.3 mm in males, *Megophrysnanlingensis* sp. nov. differs from the nine members with larger SVL values: *M.baolongensis* (42.0–45.0 mm in males), *M.binlingensis* (45.1–51.0 mm in males), *M.caudoprocta* (81.3 mm in single male), *M.jingdongensis* (53.0–56.5 mm in males), *M.latidactyla* (38.9 mm in single male), *M.omeimontis* (56.0–59.5 mm in males), *M.sangzhiensis* (54.7 mm in single male), *M.shuichengensis* (102.0–118.3 mm in males) and *M.spinata* (47.2–54.4 mm in males).

*Megophrysnanlingensis* sp. nov. differs from 12 species occurring in eastern and southern China (*M.acuta*, *M.brachykolos*, *M.boettgeri*, *M.cheni*, *M.huangshanensis*, *M.insularis*, *M.jinggangensis*, *M.kuatunensis*, *M.lini*, *M.lishuiensis*, *M.obesa* and *M.ombrophila*) by the following combination of characters: presence of vomerine teeth (vs. absent in *M.acuta*, *M.boettgeri*, *M.brachykolos*, *M.cheni*, *M.huangshanensis*, *M.kuatunensis*, *M.lini*, *M.lishuiensis*, *M.obesa* and *M.ombrophila*), margin of tongue notched posteriorly (vs. not notched in *M.acuta*, *M.brachykolos*, *M.jinggangensis*, *M.lini*, *M.lishuiensis*, *M.obesa* and *M.ombrophila*), toes with narrow lateral fringes (vs. wide in *M.boettgeri*, *M.cheni* and *M.lini*; absent in *M.brachykolos*, *M.huangshanensis*, *M.insularis*, *M.lishuiensis*, *M.obesa* and *M.ombrophila*), toes with rudimentary webbing (vs. toes without webbing in *M.lishuiensis*, *M.kuatunensis* and *M.ombrophila*), hindlimbs comparatively longer, with heels overlapping when the flexed hindlimbs are held at right angles to the body axis (vs. hindlimbs short, with heels not meeting in *M.acuta*, *M.brachykolos*, *M.huangshanensis*, *M.insularis*, *M.obesa* and *M.ombrophila*).

*Megophrysnanlingensis* sp. nov. differs from the remaining 12 members of the *Megophryss.l.* allocated to the subgenus Panophrys which share a moderate or small body size, by the small horn-like tubercle at edge of the upper eyelid (vs. horn-like tubercle indistinct or absent in *M.binchuanensis*, *M.minor*, *M.wuliangshanensis* and *M.wushanensis*; slightly large in *M.palpebralespinosa*; long point in *M.liboensis*), presence of vomerine teeth (vs. absent in *M.binchuanensis*, *M.leishanensis*, *M.minor*, *M.wuliangshanensis* and *M.wushanensis*), tongue notched posteriorly (vs. tongue not notched in *M.palpebralespinosa*, *M.tuberogranulatus* and *M.wushanensis*), toes with narrow lateral fringes (vs. wide in *M.binchuanensis*, *M.liboensis*, *M.palpebralespinosa* and *M.wushanensis* (in males)); absent in *M.daweimontis*, *M.leishanensis*, *M.minor*, *M.tuberogranulatus*, *M.wuliangshanensis*, *M.wushanensis* (in females); indistinct or absent in *M.hoanglienensis*), toes webbing rudimentary (vs. toes without webbing in *M.daweimontis*, *M.fansipanensis*, *M.rubrimera* and *M.wuliangshanensis*; indistinct or absent in *M.fansipanensis* and *M.hoanglienensis*; at least one-fourth webbed in *M.palpebralespinosa*), subarticular tubercles present (vs. absent in *M.palpebralespinosa* and *M.rubrimera*).

*Megophrysnanlingensis* sp. nov. further differs from *M.feii*, for which molecular data are lacking and cannot be allocated to any subgenus based on morphology only (Yang et al. 2018) by the larger body size, SVL 30.5–37.3 mm in males (VS. 24.3–25.1 mm in males), presence of nuptial pad with nuptial spines in males during breeding season (vs. absent), presence of vomerine teeth (vs. absent), presence of narrow lateral fringes on toes (vs. moderate or wide).

#### Description of holotype.

Adult male. Body size small, SVL 32.5 mm; head length and head width almost isometric, HDW/HDL 0.99; snout rounded in dorsal view, projecting, sloping backward to mouth in profile, protruding well beyond margin of lower jaw; top of head flat; eye large, ED/HDL 0.37, pupil vertical; nostril oblique ovoid; canthus rostralis well developed; loreal region slightly oblique; internasal distance slightly larger than interorbital distance; tympanum distinct, moderate-sized, TD/ED 0.48; large ovoid choanae at the base of the maxilla; presence of vomerine ridge bearing vomerine teeth; margin of tongue notched posteriorly; internal vocal slits present near the rear of the lower mandible.

RAD/SVL 0.25, HND/SVL 0.24; fingers without webbing and lateral fringes, relative finger length II < I < IV < III; tips of fingers slightly dilated, round; one subarticular tubercle at the bases of each finger; outer and inner metacarpal tubercles distinct, and the inner one observably enlarged. Hindlimbs slender, tibio-tarsal articulation reaching forward to the center of the eye when hindlimb is stretched along the side of the body; heels overlapping when the flexed hindlimbs are held at right angles to the body axis; TIB/SVL 0.49 and FTL/SVL 0.69; relative toe length I < II < V < III < IV; tips of toes round and slightly dilated; toes with narrow lateral fringes, rudimentary webbing; one subarticular tubercle at the bases of each toes; presence of a long ovoid inner metatarsal tubercle and absence of outer metatarsal tubercle.

Dorsal skin texture rough; head surface rough, with small tapered granules densely covering from temporal region, upper lip, loreal region to tip of snout; granules forming discontinuous X-shaped ridge with two discontinuous dorsolateral ridges on both sides at the central trunk; large tubercles on flanks; a horn-like prominent tubercle on the edge of the upper eyelid; distinct supratympanic fold curving posteroventrally from posterior corner of eye to a level above insertion of arm; ventral skin texture smooth, with several large granules and tubercles on two sides; ventral skin texture of thighs smooth, with a few small tubercles; pectoral gland larger, closer to axilla; single femoral gland on rear of thigh.

#### Measurements of holotype (in mm).

SVL 32.5, HDL 11.5, HDW 11.4, SNT 3.7, IND 3.5, IOD 3.3, ED 4.2, TD 2.0, TED 1.7, HND 8.0, RAD 7.8, FTL 22.3, TIB 15.9.

#### Coloration of holotype in life.

**Figure 8.**
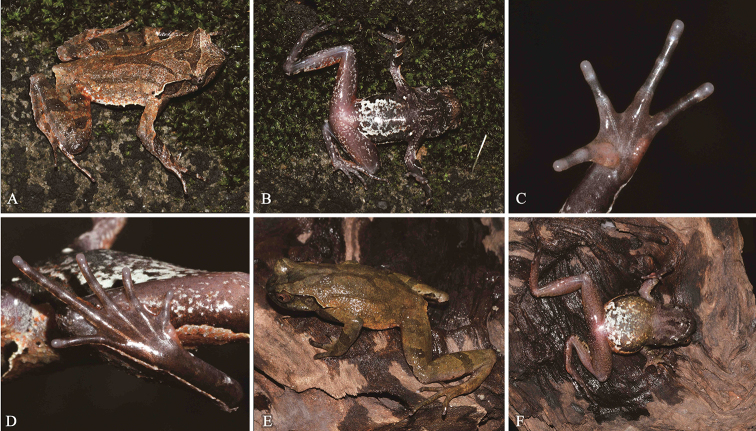
*Megophrysnanlingensis* sp. nov. in life: **A–D**SYS a001964, male holotype **E, F**SYS a001963, female paratype.

(Fig. 8A–D) Brown dorsally, with a dark triangular marking with light yellow edge between eyes, and an X-shaped marking with light yellow edge on the center of the back of trunk. Dark brown transverse bands dorsally on lower arms and hindlimbs. Surface of snout brown. Black brown vertical band below the eye on each side. Temporal region brown, supratympanic fold white. Ventral surface pale grey, an indistinct longitudinal stripe on surface of throat. Scarlet spots on surface of chest. Belly whitish grey with dark brown marbling. A pair of black longitudinal stripes scattered with several white tubercles on surface of lateroventral flanks. Ventral surface of limbs light red and scattered with white spots. Ventral surface of hands and feet dark brown, tips of digits pale-grey. Metacarpal tubercle and metatarsal tubercle light red. Pectoral glands and femoral glands white. Iris reddish brown.

#### Coloration of holotype in preservative.

Coloration of dorsal and ventral surface turned pale; transverse bands on limbs, dark longitudinal stripe on surface of throat and black patches on surface of lateroventral flanks became more distinct; scarlet spots on surface of chest faded.

#### Variation.

Measurement data of type series are listed in Table 7.

All paratypes are very similar to holotype in morphology and color pattern. However, the male specimen SYS a001963 (Fig. 8E, F) is obviously large in snout-vent length than other specimens, with lighter reddish-brown iris, yellowish brown background coloration and comparatively smooth skin. The heels are significantly overlapping in all specimens from Nanling Nature Reserve but slightly overlapping in specimens from Mt. Qiyun.

**Table 7.** Measurements (in mm; minimum-maximum, mean ± SD) of the type series of *Megophrysnanlingensis* sp. nov.

Species	*Megopgrysnanlingensis* sp. nov.
	Males (n = 10)
SVL	30.5–37.3 (33.2 ± 1.9)
HDL	10.9–12.7 (11.6 ± 0.5)
HDW	10.7–13.8 (11.8 ± 0.9)
SNT	3.4–3.8 (3.6 ± 0.1)
IND	3.5–4.0 (3.7 ± 0.2)
IOD	3.2–4.0 (3.4 ± 0.3)
ED	4.1–4.9 (4.5 ± 0.3)
TD	1.9–2.5 (2.2 ± 0.2)
TED	1.6–2.2 (1.8 ± 0.2)
HND	7.1–9.6 (8.0 ± 0.6)
RAD	7.1–9.0 (8.1 ± 0.5)
FTL	18.6–27.1 (22.4 ± 2.3)
TIB	13.9–18.8 (16.0 ± 1.3)
HDL/SVL	0.33–0.36 (0.35 ± 0.01)
HDW/SVL	0.33–0.37 (0.35 ± 0.01)
HDW/HDL	0.97–1.09 (1.02 ± 0.04)
SNT/HDL	0.30–0.33 (0.31 ± 0.01)
SNT/SVL	0.10–0.12 (0.11 ± 0.01)
IND/HDW	0.29–0.35 (0.31 ± 0.02)
IOD/HDW	0.28–0.32 (0.29 ± 0.01)
ED/HDL	0.37–0.41 (0.39 ± 0.01)
ED/SVL	0.13–0.14 (0.14 ± 0.01)
TD/ED	0.43–0.57 (0.48 ± 0.04)
TED/TD	0.67–0.95 (0.83 ± 0.10)
HND/SVL	0.23–0.26 (0.24 ± 0.01)
RAD/SVL	0.23–0.26 (0.24 ± 0.01)
TIB/SVL	0.45–0.51 (0.48 ± 0.02)
FTL/SVL	0.61–0.73 (0.68 ± 0.04)

#### Etymology.

The specific epithet “nanglingensis” is in reference to the type locality of the new species, Nanling Nature Reserve of the Nanling Mountains. We propose the common English name “Nanling Horned Toad” and Chinese name “Nan Ling Jiao Chan (南岭角蟾)”.

#### Distribution and natural history.

Currently, *Megophrysnanglingensis* sp. nov. is known from Nanling Nature Reserve and the neighboring Mangshan Nature Reserve (between elevations of 1000–1300 m), together with Mt. Qiyun (between elevations of 690–1400 m). It inhabits streams in bamboo forests. Males are frequently heard calling during August and December. Tadpoles could be found in this period.

*Megophrysnanlingensis* sp. nov. is sympatric with *M.mangshanensis* and *M.popei* in Nanling Nature Reserve and the neighboring Mangshan Nature Reserve.

### Megophrys (Panophrys) wugongensis

Taxon classificationAnimaliaAnuraMegophryidae

J. Wang, Lyu & Y.Y. Wang sp. nov.

http://zoobank.org/51EED805-C594-4FA0-A03E-9BE8C11EAA40

Fig. 9 , Table 8

#### Holotype.

SYS a002625, adult male, collected by Guo-Ling Chen and Jian Zhao on 9 May 2014 from Yangshimu Scenic Area (27°34'47.93"N, 114°15'7.34"E; 550 m a.s.l.), Pingxiang City, Jiangxi Province, China.

#### Paratypes (three males & nine females).

Adult males, SYS a004777/CIB110011, SYS a004796, 4800, collected by Zhi-Tong Lyu and Ying-Yong Wang on 23 May 2016, and adult females, SYS a002610–2611, collected by Guo-Ling Chen and Jian Zhao on 8 May 2014, SYS a004797–4799, 4801–4804, collected by Zhi-Tong Lyu and Ying-Yong Wang on 23 May 2016, from Wugongshan Scenic Area (27°34'3.94"N, 114°10'28.38"E; 1050–1080 m a.s.l.), Anfu County, Ji’an City, Jiangxi Province, China.

#### Diagnosis.

(1) Body size small, SVL 31.0–34.1 mm in four adult males and body size moderate, SVL 38.5–42.8 mm in nine adult females; (2) tympanum distinct, slightly convex, moderate-sized, TD/ED ratio 0.47–0.52; (3) vomerine teeth absent; (4) margin of tongue not notched posteriorly; (5) hindlimbs short, heels not meeting, tibia-tarsal articulation reaching forward to the region between posterior corner of eye and posterior margin of tympanum; (6) TIB/SVL ratio 0.39–0.44, FTL/SVL ratio 0.56–0.64; (7) fingers without lateral fringes, presence of a subarticular tubercle at the bases of each finger, relative finger lengths II < I = IV < III; (8) toes with rudimentary webbing at their bases and without lateral fringes, subarticular tubercles only present at the base of each toe; (9) numerous granules present on dorsal surface of body, several large tubercles present on surface of flanks and dorsal surface of limbs; (10) presence of a small horn-like tubercle at the edge of the upper eyelid; (11) supratympanic fold distinct, whitish; (12) yellowish brown or reddish brown dorsally, with an incomplete dark triangular marking between eyes and an X-shaped marking on back of trunk; (13) ventral surface greyish brown, ventral surface of abdomen with creamy white nebulous patches and black spots; (14) males with a single subgular vocal sac; (15) gravid females bear creamy yellow oocytes.

#### Comparisons.

Comparative data of *Megophryswugongensis* sp. nov. with *M.dongduanensis* sp. nov., *M.nankunensis* sp. nov., *M.jiulianensis* sp. nov., *Megophrysnanlingensis* sp. nov., *M.feii* and the 33 recognized members of Megophryss.l. allocated to thesubgenusPanophrys are listed in Table 5.

*Megophryswugongensis* sp. nov. differs from *M.dongguanensis* sp. nov., *M.nankunensis* sp. nov., *M.jiulianensis* sp. nov. and *M.nanlingensis* sp. nov. by a combination of following characters: vomerine teeth absent (vs. vomerine teeth present), tongue not notched posteriorly (vs. tongue notched in *M.jiulianensis* sp. nov. and *M.nanlingensis* sp. nov.), absence of lateral fringes on toes (vs. presence of narrow lateral fringes on toes in *M.nanlingensis* sp. nov.), heels not meeting when the flexed hindlimbs are held at right angles to the body axis (vs. heels overlapping in *M.jiulianensis* sp. nov. and *M.nanlingensis* sp. nov.), absence of black spines on dorsal skin (vs. present in *M.jiulianensis* sp. nov.), relative finger lengths II < I = IV < III (vs. II < I < IV < III in *M.nankunensis* sp. nov., *M.jiulianensis* sp. nov. and *M.nanlingensis* sp. nov.), ventral surface with creamy white nebulous patches (vs. absence of such patched on ventral surface in *M.dongguanensis* sp. nov. and *M.nankunensis* sp. nov.).

With the smaller body size, SVL 31.0–34.1 mm in males and 38.5–42.8 mm in females, *Megophryswugongensis* sp. nov. differs from the 13 members with larger SVL values: *M.baolongensis* (42.0–45.0 mm in males), *M.binlingensis* (45.1–51.0 mm in males), *M.caudoprocta* (81.3 mm in single male), *M.hoanglienensis* (37.4–47.6 mm in males, 59.6 mm in single female), *M.jingdongensis* (53.0–56.5 mm in males 63.5 in single female), *M.latidactyla* (38.9 mm in single male), *M.liboensis* (34.7–67.7 mm in males, 60.8–70.6 mm in females), *M.omeimontis* (56.0–59.5 mm in males, 68.0–72.5 mm in females), *M.palpebralespinosa* (36.2–38.0 mm in males), *M.sangzhiensis* (54.7 mm in single male), *M.shuichengensis* (102.0–118.3 mm in males, 99.8–115.6 mm in females), *M.spinata* (47.2–54.4 mm in males, 54.0–55.0 mm in females), and *M.tuberogranulatus* (33.2–39.6 mm in males, 50.5 mm in single female).

*Megophryswugongensis* sp. nov. differs from 12 species occurring in eastern and southern China (*M.acuta*, *M.brachykolos*, *M.boettgeri*, *M.cheni*, *M.huangshanensis*, *M.insularis*, *M.jinggangensis*, *M.kuatunensis*, *M.lini*, *M.lishuiensis*, *M.obesa* and *M.ombrophila*) by the following combination of characters: vomerine teeth absent (vs. present in *M.insularis* and *M.jinggangensis*), tongue not notched posteriorly (vs. tongue notched in *M.boettgeri*, *M.huangshanensis*, *M.kuatunensis* and *M.insularis*), toes without lateral fringes (vs. laterals fringes on toes narrow in *M.acuta*, *M.kuatunensis* and *M.jinggangensis*; wide in *M.boettgeri*, *M.cheni* and *M.lini*), toes with rudimentary webbing (vs. toes without webbing in *M.huangshanensis*, *M.lishuiensis* and *M.ombrophila*), hindlimbs short, with heels not meeting when the flexed hindlimbs are held at right angles to the body axis (vs. hindlimbs comparatively longer, with heels overlapping in *M.boettgeri*, *M.cheni*, *M.kuatunensis*, *M.jinggangensis* and *M .lini*), relative finger lengths II < I = IV < III (vs. I < II ≤ IV < III in *M.acuta* and *M.ombrophila*; IV < II < I < III in *M.brachykolos*; I < II = IV < III in *M.lishuiensis*; I < II ≤ IV < III in *M.obesa*), males bearing nuptial pads with nuptial spines during breeding season (vs. nuptials absence in adult males of *M.acuta*), ventral surface with creamy white nebulous patches (vs. absence of such patched in *M.brachykolos* and *M.obesa*).

*Megophrysnanlingensis* sp. nov. differs from the remaining eight members of the Megophryss.l. allocated to thesubgenusPanophrys which share a moderate or small body size, by a combination of following characters: horn-like tubercle small at edge of the upper eyelid (vs. horn-like tubercle indistinct or absent in *M.binchuanensis*, *M.minor*, *M.wuliangshanensis* and *M.wushanensis*), absence of vomerine teeth (vs. present in *M.daweimontis*, *M.fansipanensis* and *M.rubrimera*), tongue not notched posteriorly (vs. tongue notched in *M.minor*, *M.fansipanensis* and *M.rubrimera*), toes without lateral fringes (vs. lateral fringes wide in *M.binchuanensis*, *M.wushanensis* (in males); narrow in *M.rubrimera*), toes with rudimentary webbing (vs. toes without webbing in *M.daweimontis*, *M.fansipanensis*, *M.rubrimera* and *M.wuliangshanensis*), heels not meeting when the flexed hindlimbs are held at right angles to the body axis (vs. heels overlapping in *M.minor* and *M.wuliangshanensis*), heels not meeting when the flexed hindlimbs are held at right angles to the body axis (heels meeting in *M.binchuanensis*; heels meeting or overlapping in *M.minor* and *M.wushanensis*; heels overlapping in *M.leishanensis*, and *M.wuliangshanensis*).

*Megophryswugongensis* sp. nov. further differs from *M.feii*, for which molecular data are lacking and cannot be allocated to any subgenus base on morphology only (Yang et al. 2018) by the larger body size, SVL 31.0–34.1 mm in males and 38.5–42.8 mm in females (VS. 24.3–25.1 mm in males, 28.2–28.9 mm in females), presence of nuptial pad with nuptial spines in males during breeding season (vs. absent), absence of lateral fringes on toes (vs. moderate or wide).

#### Description of holotype.

Adult male. Habitus small, SVL 31.0 mm; head width slightly larger than head length, HDW/HWL 1.03; snout rounded in dorsal view, tip of snout slightly sharpened, sloping backward to mouth in profile, protruding well beyond margin of lower jaw; top of head flat; eye large, ED/HDL 0.41; nostril oblique ovoid; pupil vertical; canthus rostralis well developed; loreal region vertical; internasal distance slightly larger than interorbital distance; tympanum distinct, moderate-sized, TD/ED 0.47; large ovoid choanae at the base of the maxilla; weak vomerine ridge present, vomerine teeth absent; margin of tongue not notched posteriorly; internal vocal slits present near the rear of the lower mandible.

RAD/SVL 0.24, HND/SVL 0.22; absence of lateral fringes and webbing on fingers, relative finger lengths II < I = IV < III; tip of finger rounded, slightly swollen; presence of a distinct subarticular tubercle on the base of each finger; outer metacarpal tubercles indistinct, inner metacarpal tubercles distinct and observably enlarged. Hindlimbs short, tibio-tarsal articulation reaching forward the posterior corner of eye when hindlimb is stretched along the side of the body; heels not meeting when the flexed hindlimbs are held at right angles to the body axis; TIB/SVL 0.43 and FTL/SVL 0.61; relative toe lengths I < II < V < III < IV; tips of toes round and slightly dilated; presence of rudimentary webbing on toes but absence of lateral fringes and tarsal folds; presence of a subarticular tubercle only at the bases of each toes; presence of a long ovoid inner metatarsal tubercle and absence of outer metatarsal tubercle.

Dorsal skin texture rough with dense granules, some of which forming an X-shaped skin ridge on center of trunk; surface of flanks with large tubercles; presence of a small horn-like tubercle at the edge of eye lid; distinct supratympanic fold curving posteroventrally from posterior corner of eye to a level above insertion of arm; superior margin of tympanum in connect with supratympanic fold; ventral skin texture smooth with granules on the surface of abdomen; pectoral gland large, closer to axilla; single large femoral gland on rear of thigh.

#### Measurements of holotype (in mm).

SVL 30.8, HDL 11.9, HDW 11.7, SNT 3.5, IND 3.0, IOD 2.8, ED 3.5, TD 1.8, TED 1.7, HND 8.5, RAD 7.2, FTL 21.8, TIB 15.1

#### Coloration of holotype in life.

**Figure 9.**
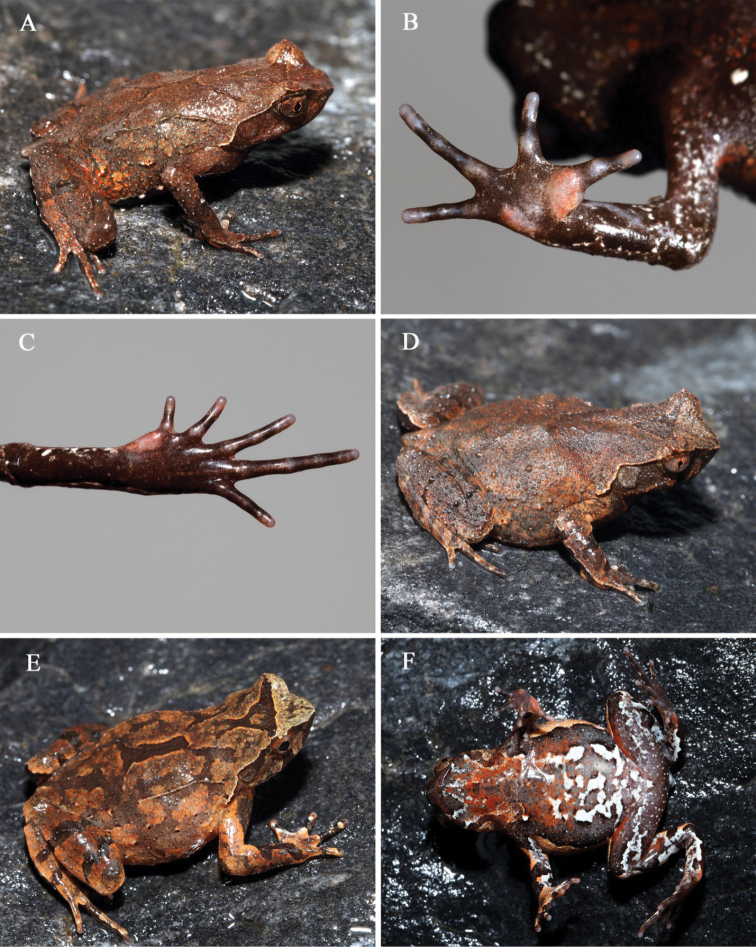
*Megophryswugongensis* sp. nov. in life: **A–C**SYS a002625, male holotype **D**SYS a002610, female paratype **E, F**SYS a002611, female paratype.

(Fig. 9A–C) Dorsal surface reddish brown, with a distinct and dark triangular marking with yellow edges between eyes. Hindlimb with broad black transverse bands. A dark brown vertical band below the eye. Canthus rostralis and supratympanic fold white. Horn-like tubercle at the edge of the upper eyelid yellow. Surface of throat and chest dark brown, with scarlet marbling, posterior region of abdomen white. Ventral surface of limbs brown with white spots and patches. Ventral surface of hand and foot brown, inner and outer metatarsal tubercles and inner metacarpal tubercle pink. Pectoral and femoral glands white. Iris reddish brown.

#### Coloration of holotype in preservative.

Dorsum dark brown, markings on dorsal surface became indistinct, transverse bands on limbs became dark grey and became more distinct. Surface of throat and chest light brown, posterior region of abdomen light yellow, ventral surface of limbs light brown, inner and outer metatarsal tubercles and inner metacarpal tubercle light yellow, all marbling, colored spots and patches absent.

#### Variation.

Measurement data of type series are listed in Table 8.

All paratypes are very similar to holotype in morphology and color pattern. However, dorsal surface yellowish brown in female paratypes SYS a004798, 4801, 4804, markings on dorsal skin indistinct in male paratypes SYS a004777/CIB110011 and SYS a004796, and female paratypes SYS a002610 (Fig. 9D), 4797, 4799, presence of a rectangle marking on central back of trunk in the female paratype SYS a002611 (Fig. 9E–F).

**Table 8.** Measurements (in mm; minimum-maximum, mean ± SD) of the type series of *Megophryswugongensis* sp. nov.

Species	*Megophryswugongensis* sp. nov.	
	Males (n = 4)	Females (n = 9)
SVL	31.0–34.1 (32.4 ± 1.3)	38.5–42.8 (40.8 ± 1.3)
HDL	10.2–11.2 (10.7 ± 0.4)	11.8–13.2 (12.6 ± 0.4)
HDW	10.4–11.9 (11.0 ± 0.6)	12.6–13.9 (13.4 ± 0.4)
SNT	3.4–3.9 (3.8 ± 0.2)	4.2–4.8 (4.6 ± 0.2)
IND	3.6–3.7 (3.7 ± 0.1)	3.6–4.2 (4.0 ± 0.2)
IOD	3.1–3.4 (3.2 ± 0.1)	3.6–3.8 (3.7 ± 0.1)
ED	4.1–4.4 (4.3 ± 0.1)	4.1–5.1 (4.4 ± 0.3)
TD	2.0–2.2 (2.1 ± 0.1)	2.1–2.3 (2.2 ± 0.1)
TED	1.7–2.2 (1.9 ± 0.2)	2.1–2.6 (2.4 ± 0.2)
HND	6.5–7.3 (7.0 ± 0.3)	8.2–9.7 (8.7 ± 0.5)
RAD	6.7–7.8 (7.4 ± 0.5)	8.1–9.8 (8.9 ± 0.6)
FTL	17.8–20.9 (19.2 ± 1.3)	21.8–25.0 (23.3 ± 1.1)
TIB	12.4–14.3 (13.3 ± 0.8)	15.0–17.9 (16.0 ± 0.9)
HDL/SVL	0.31–0.34 (0.33 ± 0.01)	0.30–0.33 (0.31 ± 0.01)
HDW/SVL	0.32–0.36 (0.34 ± 0.02)	0.32–0.35 (0.33 ± 0.01)
HDW/HDL	1.01–1.06 (1.03 ± 0.02)	1.03–1.08 (1.06 ± 0.02)
SNT/HDL	0.32–0.37 (0.35 ± 0.02)	0.33–0.40 (0.36 ± 0.02)
SNT/SVL	0.11–0.12 (0.12)	0.11–0.12 (0.11 ± 0.01)
IND/HDW	0.31–0.35 (0.33 ± 0.02)	0.27–0.32 (0.30 ± 0.02)
IOD/HDW	0.27–0.31 (0.29 ± 0.02)	0.27–0.30 (0.28 ± 0.01)
ED/HDL	0.37–0.41 (0.40 ± 0.02)	0.31–0.40 (0.35 ± 0.03)
ED/SVL	0.13–0.14 (0.13 ± 0.01)	0.10–0.13 (0.11 ± 0.01)
TD/ED	0.47–0.52 (0.49 ± 0.02)	0.45–0.53 (0.51 ± 0.03)
TED/TD	0.85–1.10 (0.92 ± 0.12)	0.91–1.14 (1.09 ± 0.07)
HND/SVL	0.20–0.22 (0.21 ± 0.01)	0.20–0.23 (0.21 ± 0.01)
RAD/SVL	0.21–0.24 (0.23 ± 0.02)	0.20–0.25 (0.22 ± 0.02)
TIB/SVL	0.39–0.44 (0.41 ± 0.02)	0.37–0.44 (0.39 ± 0.02)
FTL/SVL	0.56–0.64 (0.59 ± 0.04)	0.54–0.60 (0.57 ± 0.02)

#### Etymology.

The specific epithet “wugongensis” is in reference to the type locality of the new species in the Wugong Mountains. We propose the common English name “Wugongshan Horned Toad” and Chinese name “Wu Gong Shan Jiao Chan (武功山角蟾)”.

#### Distribution and habits.

Currently, *Megophryswugongensis* sp. nov. is known from the type locality, Yangshimu Scenic Area, Pingxiang City, Jiangxi Province at approximate 550 m a.s.l., Wugongshan Scenic Area, Ji’an City, Jiangxi Province at approximate 1050–1080 m a.s.l., all located in the Luoxiao Mountains in eastern China. All specimens were collected on leaf litter near a stream in the bamboo forest, males were not heard calling. In consideration of the invisible nuptial pad and nuptial spines in all male specimens and the undeveloped fallopian tubes in all female specimens, the breeding season of *M.wugongensis* sp. nov. still remains unknown. Tadpoles were not observed. *Megophryswugongensis* sp. nov. is sympatric with *M.jinggangensis* in all localities.

### Megophrys (Panophrys) mufumontana

Taxon classificationAnimaliaAnuraMegophryidae

J. Wang, Lyu & Y.Y. Wang sp. nov.

http://zoobank.org/4FD2EE4D-A6D7-4F72-896C-A3866D74DFB7

Fig. 10 , Table 9

#### Holotype.

SYS a006391, adult male, collected by Zhi-Tong Lyu on 3 August 2017 from Mt. Mufu (28°58'18.45"N, 113°48'58.53"E; 1300 m a.s.l.), Pingjiang County, Yueyang City, Hunan Province, China.

#### Paratypes (one male & two females).

Adult females, SYS a006390/CIB110012, SYS a006419, and the other adult male, SYS a006392, all collected by Zhi-Ting Lyu on 3 August 2017 from the same locality as the holotype.

#### Diagnosis.

(1) Body size small, SVL 30.1–30.8 mm in two adult males and SVL 36.3 mm in two adult females; (2) head length slightly larger than head width, HDW/HDL ratio 0.98–0.99; (3) tympanum distinct, moderate-sized, TD/ED ratio 0.51–0.58, upper 1/4 part of the tympanum concealed by supratympanic fold; (4) vomerine teeth absent; (5) margin of tongue not notched posteriorly; (6) heels overlapping, tibia-tarsal articulation reach forward to the tympanum in males and to the eye in females; (7) TIB/SVL ratio 0.47–0.53, FTL/SVL ratio 0.68–0.74; (8) fingers without lateral fringes, presence of a subarticular tubercle at the bases of each finger, relative finger lengths II = IV < I < III; (9) toes with rudimentary webbing at their bases and narrow lateral fringes, subarticular tubercles only present at the base of each toe; (10) numerous granules scattered with tubercles present on dorsal surface of body, limbs and surface of flanks, some of which forming a V-shaped, \ /-shaped or X-shaped skin ridge on central back of trunk; (11) presence of a small horn-like tubercle at the edge of the upper eyelid; (12) supratympanic fold distinct; (13) light brown to dark brown dorsally, with a dark triangular marking between eyes; (14) a pair of dark longitudinal and irregular marking with white edges on its upper side on ventrolateral surface of flanks; (15) surface of throat and chest greyish brown with dark brown patches and creamy white spots, surface of abdomen greyish white with creamy white and orange spots; (16) ventral surface of thighs with dense small whitish tubercles.

#### Comparisons.

Comparative data of *Megophrysmufumontana* sp. nov. with *M.dongduanensis* sp. nov., *M.nankunensis* sp. nov., *M.jiulianensis* sp. nov., *Megophrysnanlingensis* sp. nov., *Megophryswugongensis* sp. nov., *M.feii* and the 33 recognized members of Megophryss.l. allocated to thesubgenusPanophrys are listed in Table 5.

*Megophrysmufumontana* sp. nov. differs from *M.dongguanensis* sp. nov., *M.nankunensis* sp. nov., *M.jiulianensis* sp. nov. and *M.wugongensis* sp. nov. by upper 1/4 part of the tympanum concealed by supratympanic fold (vs. tympanum entirely visible), the heels overlapping when hindlimb is stretched along the side of the body (vs. heels not meeting in *M.dongguanensis* sp. nov., *M.nankunensis* sp. nov., *M.jiulianensis* sp. nov. and *M.wugongensis* sp. nov.), presence of narrow lateral fringes on toes (vs. absent in *M.dongguanensis* sp. nov., *M.nankunensis* sp. nov. and *M.jiulianensis* sp. nov.), absence of vomerine teeth (vs. present in *M.dongguanensis* sp. nov., *M.nankunensis* sp. nov., *M.jiulianensis* sp. nov. and *M.nanlingensis* sp. nov.), tongue not notched posteriorly (vs. tongue notched in *M.jiulianensis* sp. nov. and *M.nanlingensis* sp. nov.), skin relatively smooth and lacking black horny spines (vs. skin rough with black horny spines in *M . jiulianensis* sp. nov.).

With the smaller body size, SVL 30.1–30.8 mm in males and 36.3 mm in females, *Megophrysmufumontana* sp. nov. differs from the 19 members with larger SVL values: *M.baolongensis* (42.0–45.0 mm in males), *M.binchuanensis* (32.0–36.0 mm in males, 40.2–42.5 mm in females), *M.binlingensis* (45.1–51.0 mm in males), *M.caudoprocta* (81.3 mm in single male), *M.daweimontis* (34.0–37.0 mm in males, 40.0–46.0 mm in females), *M.fansipanensis* (41.7–42.5 mm in females), M.hoanglienensis (37.4–47.6 mm in males, 59.6 mm in single female), *M.jingdongensis* (53.0–56.5 mm in males 63.5 in single female), *M.latidactyla* (38.9 mm in single male), *M.liboensis* (34.7–67.7 mm in males, 60.8–70.6 mm in females), *M.minor* (34.5–41.2 mm in males), *M.omeimontis* (56.0–59.5 mm in males, 68.0–72.5 mm in females), *M.palpebralespinosa* (36.2–38.0 mm in males), *M.sangzhiensis* (54.7 mm in single male), *M.shuichengensis* (102.0–118.3 mm in males, 99.8–115.6 mm in females), *M.spinata* (47.2–54.4 mm in males, 54.0–55.0 mm in females), *M.tuberogranulatus* (33.2–39.6 mm in males, 50.5 mm in single female), *M.wushanensis* (38.4 mm in single female) and *M.wuliangshanensis* (41.3 mm in single female).

*Megophrysmufumontana* sp. nov. differs from 12 species occurring in eastern and southern China (*M.acuta*, *M.brachykolos*, *M.boettgeri*, *M.cheni*, *M.huangshanensis*, *M.insularis*, *M.jinggangensis*, *M.kuatunensis*, *M.lini*, *M.lishuiensis*, *M.obesa* and *M.ombrophila*) by the following combination of characters: upper 1/4 part of the tympanum concealed by supratympanic fold (vs. tympanum entirely visible in the 12 species above), absence of vomerine teeth (vs. present in *M.insularis* and *M.jinggangensis*), tongue not notched posteriorly (vs. tongue notched in *M.boettgeri*, *M.cheni*, *M.huangshanensis*, *M.insularis* and *M.kuatunensis*), presence of narrow lateral fringes on toes (vs. absent in *M.brachykolos*, *M.huangshanensis*, *M.insularis*, *M.lishuiensis*, *M.obesa* and *M.ombrophila*; wide in *M.boettgeri* and *M.cheni*), toes with rudimentary webbing (vs. toes without webbing in *M.huangshanensis*, *M.kuatunensis, M.lishuiensis* and *M.ombrophila*), the heels overlapping when hindlimb is stretched along the side of the body (vs. heels not meeting in *M.acuta*, *M.brachykolos*, *M.insularis, M.obesa* and *M.ombrophila*).

*Megophrysmufumontana* sp. nov. differs from the remaining *M.leishanensis* and *M.rubrimera* allocated to the subgenus Panophrys by the absence of vomerine teeth (vs. present in *M.rubrimera*), tongue not notched posteriorly (vs. tongue notched in *M.rubrimera*), upper 1/4 part of the tympanum concealed by supratympanic fold (vs. tympanum entirely visible in *M.leishanensis* and *M.rubrimera*), toes with narrow lateral fringes (vs. absent in *M.leishanensis*; indistinct or absent in *M.rubrimera*).

*Megophrysmufumontana* sp. nov. further differs from *M.feii*, for which molecular data are lacking and cannot be allocated to any subgenus base on morphology only (Yang et al. 2018) by the larger body size, SVL 30.1–30.8 mm in males and 36.3 mm in females (VS. 24.3–25.1 mm in males, 28.2–28.9 mm in females), tongue not notched posteriorly (vs. tongue notched), toes with narrow lateral fringes (vs. moderate or wide).

#### Description of holotype.

Adult male. Habitus small, SVL 30.8 mm; head length slightly larger than head width, HDW/HWL 0.98; snout rounded in dorsal view, sloping backward to mouth in profile, protruding well beyond margin of lower jaw; top of head flat; eye large, ED/HDL 0.30; nostril oblique ovoid; pupil vertical; canthus rostralis well developed; loreal region vertical; internasal distance slightly larger than interorbital distance; tympanum distinct, moderate-sized, TD/ED 0.56; large ovoid choanae at the base of the maxilla; weak vomerine ridge present, vomerine teeth absent; margin of tongue not notched posteriorly.

RAD/SVL 0.25, HND/SVL 0.30; absence of lateral fringes and webbing on fingers, relative finger lengths II = IV < I < III; tip of finger rounded, slightly swollen; presence of a distinct subarticular tubercle on the base of each finger; outer metacarpal tubercles indistinct, inner metacarpal tubercles distinct and observably enlarged. Hindlimbs long, tibio-tarsal articulation reaching forward to the tympanum when hindlimb is stretched along the side of the body; heels overlapping when the flexed hindlimbs are held at right angles to the body axis; TIB/SVL 0.53 and FTL/SVL 0.74; relative toe lengths I < II < V < III < IV; tips of toes round and slightly dilated; presence of rudimentary webbing and narrow lateral fringes on toes but absence of tarsal folds; presence of a subarticular tubercle only at the bases of each toes; presence of a long ovoid inner metatarsal tubercle and absence of outer metatarsal tubercle.

Dorsal skin texture rough with dense granules and scattered with small tubercles, some of which forming a \ /-shaped skin ridge on central back of trunk; presence of a small horn-like tubercle at the edge of upper eye lid; distinct supratympanic fold curving posteroventrally from posterior corner of eye to a level above insertion of arm; upper 1/4 part of the tympanum covered by supratympanic fold; ventral skin texture smooth with granules; pectoral gland large, closer to axilla; single large femoral gland on rear of thigh.

#### Measurements of holotype (in mm).

SVL 30.1, HDL 11.6, HDW 11.4, SNT 3.5, IND 3.0, IOD 2.8, ED 3.5, TD 18, TED 1.7, HND 8.5, RAD 7.2, FTL 21.8, TIB 15.1.

#### Coloration of holotype in life.

**Figure 10.**
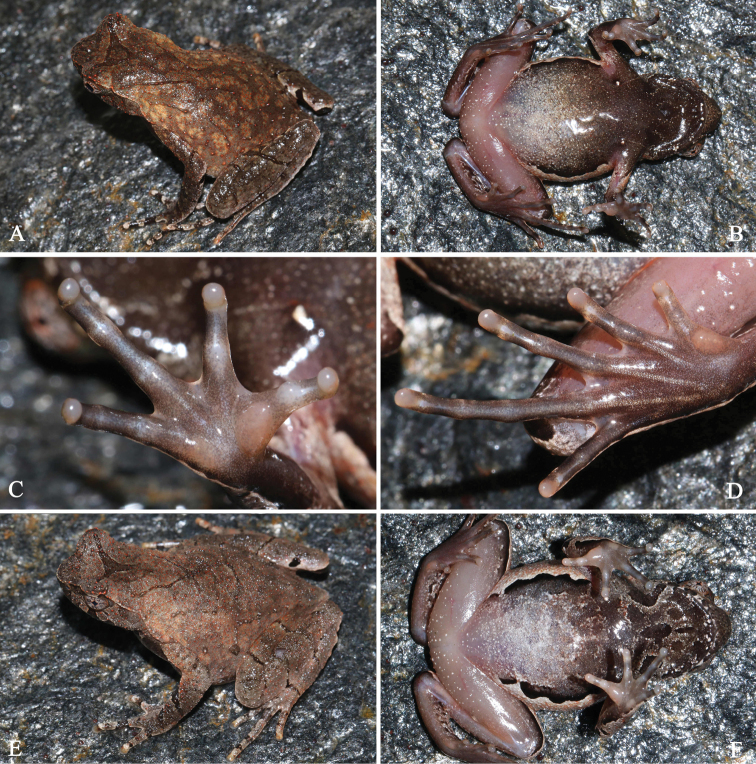
*Megophrysmufumontana* sp. nov. in life: **A–D**SYS a006391, male paratype **E–F**SYS a006392, female paratype.

(Fig. 10A–D) Dorsal surface brown, with a distinct and incomplete dark triangular marking between eyes. Hindlimb with black transverse bands. A dark brown vertical band below the eye. Horn-like tubercle at the edge of the upper eyelid red. Surface of throat and chest greyish brown with dark brown patches. Surface of abdomen greyish white with creamy white and orange spots. Ventral surface of limbs pink with white spots and light-yellow patches. Ventral surface of hand and foot brown, inner and outer metatarsal tubercles and inner metacarpal tubercle pink. Pectoral and femoral glands white. Iris white.

**Coloration of holotype in preservative.** Coloration of dorsum dark brown, markings on dorsal surface and transverse bands on limbs became indistinct. Ventral surface of throat, chest and abdomen dark grey. All patches on ventral surface indistinct, all colored spots absent. Ventral surface of limbs light yellow.

#### Variation.

Measurement data of type series are listed in Table 9.

All paratypes are very similar to holotype SYS a006391 in morphology and color pattern. However, tibia-tarsal articulation reaching forward to the eye when hindlimb is stretched along the side of the body in all females, and granules and tubercles forming a \ /-shaped skin ridge on central back of trunk in the holotype (vs. X-shaped in SYS a006390, 6419; V-shaped in SYS a006392 (Fig. 10E–F)).

**Table 9.** Measurements (in mm; minimum-maximum, mean ± SD) of the type series of *Megophrysmufumontana* sp. nov.

Species	*Megophrysmufumontana* sp. nov.	
	Males (n = 2)	Females (n = 2)
SVL	30.1–30.8	36.3
HDL	11.6–11.9	11.8–12.4
HDW	11.4–11.7	11.7–12.3
SNT	3.5–3.7	3.7–4.2
IND	3.0–3.1	3.5–3.6
IOD	2.8–2.9	3.2–3.3
ED	3.5–3.6	3.7–3.8
TD	1.7–1.8	2.1–2.2
TED	1.7–1.8	1.8–1.9
HND	8.5–9.2	9.4–9.9
RAD	7.2–7.7	8.0–8.2
FTL	21.8–22.9	24.8–25.1
TIB	15.1–16.3	16.9–17.5
HDL/SVL	0.39	0.33–0.34
HDW/SVL	0.38	0.32–0.34
HDW/HDL	0.98	0.99
SNT/HDL	0.30–0.31	0.31–0.34
SNT/SVL	0.12	0.10–0.12
IND/HDW	0.26	0.29–0.30
IOD/HDW	0.25	0.27
ED/HDL	0.30	0.31
ED/SVL	0.12	0.10
TD/ED	0.51–0.56	0.57–0.58
TED/TD	0.90–0.94	0.86
HND/SVL	0.28–0.30	0.26–0.27
RAD/SVL	0.24–0.25	0.22–0.23
TIB/SVL	0.50–0.53	0.47–0.48
FTL/SVL	0.72–0.74	0.68–0.69

#### Etymology.

The specific epithet “mufumontana” is in reference to the type locality of the new species, Mt. Mufu. We propose the common English name “Mufushan Horned Toad” and Chinese name “Mu Fu Shan Jiao Chan (幕阜山角蟾)”.

#### Distribution and habits.

Currently, *Megophrysmufumontana* sp. nov. is known only from Mt. Mufu, Pingjiang County, Yueyang City, Hunan Province, China at approximate 1300 m a.s.l.. All specimens were collected on leaf litter near a stream (about 5 m wide) surrounded by moist subtropical evergreen broadleaved forests, males were not heard calling. Tadpoles were not observed. Because none of the males have nuptial pads developed and none of the females have fallopian tubes and eggs developed, the breeding season of *M.mufumontana* sp. nov. remains unknown.

## Discussion

*Megophrysdongguanensis* sp. nov. is easily confused with *M.brachykolos* because of the relatively short shanks. In addition, the type locality of the new species is at a straight-line distance of approximately 72 km from the type locality (Hongkong Island), and at a straight-line distance of approximately 32 km from the closest locality (Sanzhoutian of Shenzhen City) of *M.brachykolos*. Currently, eight Megophrysspecies in thesubgenusPanophrys were found to have comparatively short shanks with heels not meeting when thighs are adpressed at right angles with respect to the body axis: *M.dongguanensis* sp. nov., *M.nankunensis* sp. nov., *M.wugongensis* sp. nov., *M.acuta*, *M.brachykolos*, *M.insularis*, *M.megacephala* and *M.obesa*.

In our previous study (Liu et al. 2018), 41 cryptic species within the subgenus Panophrys were revealed, and one of them was recently described as *Megophrysleishanensis* by Li et al. (2018). Moreover, except for *M.mufumontana* sp. nov. (not mentioned in Liu et al. (2018)), five of them are described in this study. Currently, the total number of recognized species of the subgenus Panophrys rises to 39, which makes it the most species-rich subgenus of *Megophrys* (≈46.4%). It’s worth noting that there remain still 33 undescribed species according to Liu et al. (2018), and 27 of them are found in southeastern China, which further reveals the unusually high level of species diversity in this region.

As the diversity of the subgenus Panophrys was confirmed to be extremely underestimated (Chen et al. 2017; Mahony et al. 2017; Liu et al. 2018), a number of new *Panophrys* species have been described since 2017 (i.e. *Megophryslishuiensis*, *M.insularis*, *M.rubrimera*, *M.liboensis*, and six new species in this study). However, all of these species have narrow distributions. For example, *M.insularis* is currently known only from an offshore island in Guangdong (Wang et al. 2017b), and *M.liboensis* is currently known only from a cave in Libo, Guizhou (Zhang et al. 2017). For the six new species in this study, *M.dongguanensis* sp. nov., *M.nankunensis* sp. nov., *M.wugongensis* sp. nov. and *M.mufumontana* sp. nov. are currently only found in their type localities. This situation of “micro-endemism” (Liu et al. 2018) has brought great challenges for the protection of these unique toads.

Among the six new species described in this paper, *M.jiulianensis* sp. nov. is sympatric with *M.nankunensis* sp. nov. in Mt. Nankun while also being sympatric with *M.hongshanensis* sp. nov. in Mt. Jiulian. Further, *M.mufumontana* sp. nov. is sympatric with a known congener *M.jinggangensis* in Mt. Mufu and *M.wugongensis* sp. nov. is sympatric with *M.jinggangensis* in Mt. Wugong. By combining the localities of these species in our phylogenetic trees (Fig. 2), our results also support the conclusion of “sympatric but distant phylogenetically” (Liu et al. 2018), that is, sympatric distribution is very common in horned toads within the subgenus Panophrys while they are distantly related in the phylogeny (Fei et al. 2012; Li et al. 2014; Wang et al. 2014; Liu et al. 2018). These geographical patterns of “sympatric but distant phylogenetically” and “micro-endemism” indicate that the Asian horned toads would be good candidates for studies on speciation and biogeography.

## Supplementary Material

XML Treatment for Megophrys (Panophrys) dongguanensis

XML Treatment for Megophrys (Panophrys) nankunensis

XML Treatment for Megophrys (Panophrys) jiulianensis

XML Treatment for Megophrys (Panophrys) nanlingensis

XML Treatment for Megophrys (Panophrys) wugongensis

XML Treatment for Megophrys (Panophrys) mufumontana

## Appendix 1

**Specimens of comparative species examined**

*Megophrysboettgeri* (n = 13): China: Jiangxi Provence: Guixi City: Yangjifeng Nature Reserve (the middle area of Wuyi Mountains, 600–883 m a.s.l.): SYS a000312, 000315, 000328–000330, 000376, 000378; Guangfeng County: Tongboshan Nature Reserve (the eastern area of Wuyi Mountains, 450–821 m a.s.l.): SYS a001671–001673, 001683, 001700.

*Megophrysbrachykolos* (n = 21): China: Hong Kong: SYS a001502–001503; Guangdong Province: Shenzhen City: Yangtaishan Forest Park (60–150 m a.s.l.): SYS a2051–002056, 002069–002074, 002413; Qiniangshan Geological Park (30–50 m a.s.l.): SYS a002405–002410.

*Megophryscaudoprocta* (n = 3): China: Hunan Province: Zhangjiajie City: Sangzhi County: Mt. Badagong (1100–1200 m a.s.l.): SYS a004281, 004308–4309.

*Megophryscheni* (n = 19): China: Jiangxi Province: Jinggangshan City: Mt. Jinggang (1200–1260 m a.s.l.): SYS a001427–001429, SYS a001871–001873; Hunan Province: Yanling County: Taoyuandong Nature Reserve: Lishuzhou Village (1480–1530 m a.s.l.): SYS a002123–002127, Dayuan Farm (1480 m a.s.l.): SYS a002140–002145.

*Megophryshuangshanensis* (n = 10): China: Jiangxi Province: Wuyuan County: Mount Dazhang (600–900 m a.s.l.): SYS a001314–001323.

*Megophrysinsularis* (n = 6): China: Guangdong Province: Shantou City: Nan’ao Island (50–500 m a.s.l.): SYS a002167–002171, SYS a003666/CIB 106881.

*Megophrysjingdongensis* (n = 2): Yunnan Province: Jingdong County: Mt. Wuliang (1800 m a.s.l.): SYS a003928–3929.

*Megophrysjinggangensis* (n = 10): China: Jiangxi Province: Jinggangshan City: Mt. Jinggang (700–900m a.s.l.): SYS a001413–001416, 001430; Hunan Province: Yanling County: Taoyuandong Nature Reserve (800–1000 m a.s.l.): SYS a001859–001863.

*Megophryskuatunensis* (n = 3): China: Fujian Province: Wuyishan City (=Ch’ungan Hsien): Guadun Village (= Kuatun Village, 1060–1220 m a.s.l.): SYS a001579 and 001590; Jiangxi Province: Guixi City: Yangjifeng Nature Reserve (the middle area of Wuyi Mountains, 950 m a.s.l.): SYS a000241.

*Megophryslini* (n = 27): China: Jiangxi Province: Jinggangshan City: Mt. Jinggang (1100–1610 m a.s.l.): SYS a001417–001424, SYS a002375–002386; Suichuan County: Nanfengmian Nature Reserve (1150–1250 m a.s.l.): SYS a002369–002374; Hunan Province: Yanling County: Taoyuandong Nature Reserve: Niushiping Village (1360 m a.s.l.): SYS a002128.

*Megophrysminor* (n = 4): China: Sichuan Province: Dujiangyan City: Mt. Qingcheng: SYS a003209, 003211–3213.

*Megophrysomeimontis* (n = 6): China: Sichuan Province: Mt. Emei: SYS a001798–001801, 001940–001941.

*Megophryssangzhiensis* (n = 6): China: Hunan Province: Zhangjiajie City: Sangzhi County: Mt. Badagong (1100–1200 m a.s.l.): SYS a004306–004307, 004313–004316.

*Megophrysspinata* (n = 2): China: Guizhou Province: Leishan County: Mt. Leigong: SYS a002226–002227.

*Megophrystuberogranulatus* (n = 1): China: Hunan Province: Zhangjiajie City: Sangzhi County: Mt. Badagong (1100–1200 m a.s.l.): SYS a004310.*Megophryswuliangshanensis* (n = 2): Yunnan Province: Jingdong County: Mt. Wuliang (1800 m a.s.l.): SYS a003924–3925.
